# Real-Time Detection and Visualization of Amyloid-β Aggregates Induced by Hydrogen Peroxide in Cell and Mouse Models of Alzheimer’s Disease

**DOI:** 10.1021/acsami.2c07859

**Published:** 2022-07-22

**Authors:** Xueli Wang, Ashok Iyaswamy, Di Xu, Senthilkumar Krishnamoorthi, Sravan Gopalkrishnashetty Sreenivasmurthy, Yuncong Yang, Yinhui Li, Chen Chen, Min Li, Hung-Wing Li, Man Shing Wong

**Affiliations:** † Department of Chemistry, Hong Kong Baptist University, 224 Waterloo Road, Kowloon Tong, Hong Kong, SAR 00000, China; ‡ Mr. & Mrs. Ko Chi-Ming Centre for Parkinson’s Disease Research, School of Chinese Medicine, Hong Kong Baptist University, 7 Baptist University Road, Kowloon Tong, Hong Kong, SAR 00000, China; ∥ Department of Chemistry, The Chinese University of Hong Kong, Room 243, Science Centre, North Block, Shatin, Hong Kong, SAR 00000, China; ⊥ Centre for Trans-disciplinary Research, Department of Pharmacology, Saveetha Dental College and Hospitals, 162, Poonamallee High Road, Chennai, Tamil Nadu 600077, India

**Keywords:** H_2_O_2_-responsive fluorescent probe, ratiometric imaging, Aβ-enhanced sensitivity, Aβ-targeting, in-vivo detection, Alzheimer’s disease

## Abstract

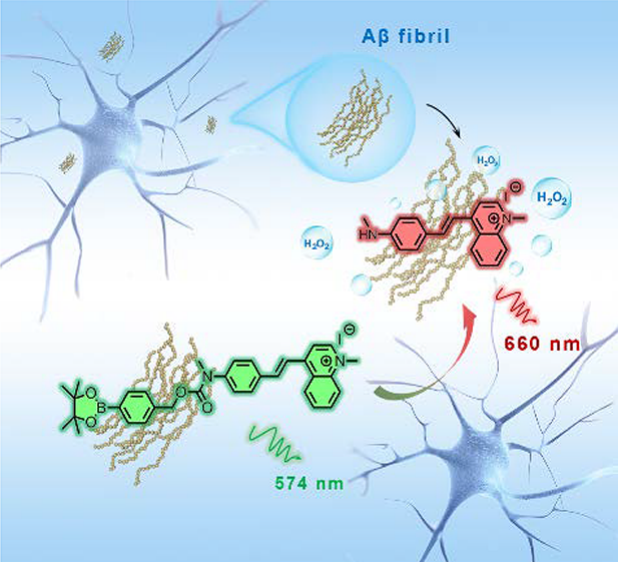

Oxidative stress, caused by an imbalance between the production and the accumulation of reactive oxygen species (ROS), is a prominent cause of the neurotoxicity induced by aggregated amyloid-β (Aβ) in Alzheimer’s disease (AD). Tools that can directly detect and monitor the presence and amount of Aβ-induced ROS are still lacking. We report herein the first Aβ-targeted ratiometric H_2_O_2_-responsive fluorescent probe for real-time detection and monitoring of the Aβ-induced H_2_O_2_ level in cell and AD mouse models. The H_2_O_2_-responsive probe is constructed based on a methylamino-substituted quinolinium-based cyanine as the fluorescence moiety and a phenylboronate ester as the sensing reaction site. This sensing probe exhibits a large emission wavelength shift of ∼87 nm upon reacting with H_2_O_2_, a high binding selectivity for Aβ, and a faster response toward H_2_O_2_ in the presence of Aβ, concomitant with an enhanced fluorescence intensity, hence greatly boosting the sensitivity of in-situ H_2_O_2_ detection. This biocompatible and nontoxic probe is capable of ratiometrically detecting and imaging endogenous H_2_O_2_ induced by Aβ in a neuronal cell model. Remarkably, this Aβ-targeted H_2_O_2_-responsive probe is also able to detect, monitor, and differentiate different Aβ-induced H_2_O_2_ levels in real time in different age groups of transgenic AD mice in which the cerebral H_2_O_2_ level increases age dependently concomitant with the plaque contents. Therefore, this smart probe can act as a powerful tool to diagnose high-risk subjects and diseased brains of AD and to further study the role of ROS in AD pathology.

## 1 Introduction

Alzheimer’s disease (AD) is the most prevalent cause of neurodegenerative disorder, and it is characterized by progressive behavior and personality changes, memory loss, and cognitive impairment.^1^ There are three characteristic pathological changes in AD: abnormal deposition of amyloid plaques, mainly comprised of amyloid-β (Aβ) peptides between brain cells and neurofibrillary tangles (NFT), primarily consisting of hyperphosphorylated tau proteins inside neurons; significant synaptic degeneration and reductions in synapses; and progressive neuronal loss.^2^ Current diagnostic methods for AD based on either behavioral changes and cognitive impairment or the detection of senile plaques by PET can only be diagnosed in the advanced stage of AD.^3,4^ Although medications can be applied for symptomatic relief, there are currently no curable treatment options to stop or reverse the disease. Therefore, there is a compelling need to identify a reliable biomarker and to develop effective and sensitive detection tools for the early diagnosis of AD so that delaying measures and early interventions can be implemented before the onset of symptomatic disease. Such a tool will also be useful to understand AD pathogenesis and progression and to evaluate drug development strategies and potential therapeutics.

The close relationship between oxidative stress caused by excess production of reactive oxygen species (ROS) relative to existing antioxidants and AD has recently attracted attention.^5−7^ The brain is highly susceptible to oxidative stress damage due to its intrinsically low antioxidant capacity, particularly against progressive oxidative damage with age. The interplay between oxidative stress and inflammation activates Aβ production and increases in Aβ deposition, which in turn gives rise to a series of pathological changes in the brain, including the formation of NFT, inflammation, and mitochondrial dysfunction, aggravating oxidative stress, and ultimately resulting in neuronal cell death and dementia.^8^ In addition, studies have shown that Aβ plaques are surrounded by high levels of ROS, such as H_2_O_2,_ in the brain samples of AD patients and transgenic (Tg) mice, implicating that the oxidative stress induced by misfolded Aβ aggregates plays a vital role in the pathogenesis of AD, which occurs in the initial stage of mild cognitive impairment in AD.^9−11^ This strongly suggests that ROS represent an important biomarker for early diagnosis and a therapeutic target for the development of AD drugs.

Fluorescent probes with near-infrared (NIR) emission are attractive and useful for bioapplications owing to their low light scattering, deep tissue penetration, minimal photodamage, and weak autofluorescence interference from complex biological systems.^12−15^ Compared to a single-emission wavelength measurement, a ratiometric detection approach with an intrinsic self-calibration function for the correction of various analyte-independent factors has been found to be particularly intriguing for bioimaging and sensing as it can provide more reliable and accurate analyses. Despite numerous probes that have been developed for the detection of ROS in cells and organs,^16−21^ effective and highly selective probes for the real-time detection and monitoring of specific ROS in AD brains in vivo are rare, particularly for the detection of H_2_O_2_ (Table S3) due to its slow reaction kinetics. Ran and co-workers developed the ROS-responsive oxalate–curcumin-based imaging probe CRANAD-61 in which its emission wavelength shifted from 810 to 570 nm upon reacting with various ROS. This probe was shown to be able to image ROS at the microlevel by two-photon imaging and at the macrolevel of total ROS content in the brains of an AD mouse model.^22^ James and co-workers reported several 3-hydroxyflavone boronate-based ratiometric fluorescent probes that could be used for in-vitro detection of reactive nitrogen species, peroxynitrite (ONOO^–^), while bound to Aβ aggregates in the brain sections of a transgenic mouse.^23^

**Figure 1:**
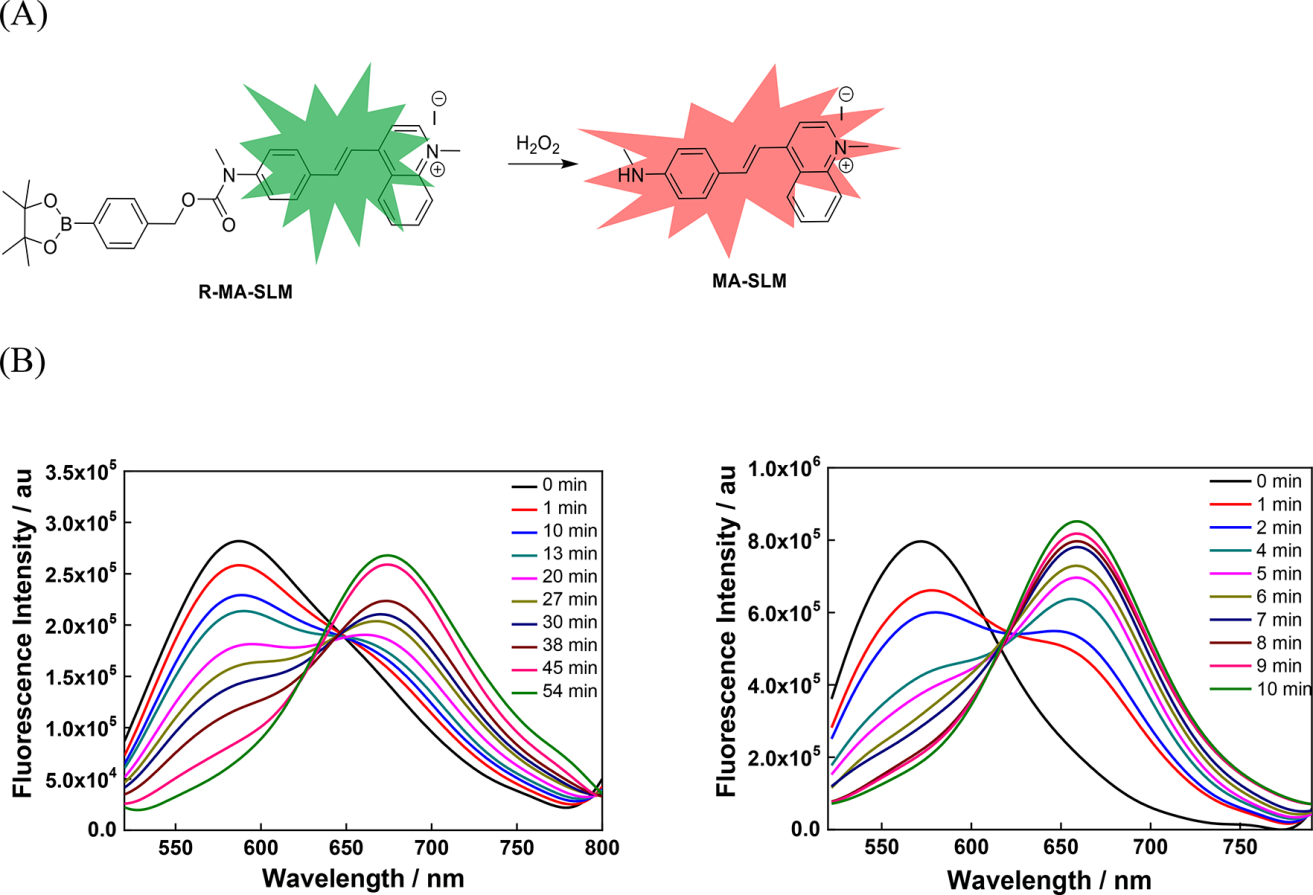
(A) Reaction of R-MA-SLM upon addition of H_2_O_2,_ affording the oxidized product MA-SLM. (B) Fluorescence spectra of R-MA-SLM (20 μM) in the absence (left) and presence (right) of Aβ_1–40_ fibril (10 μM) in PBS with respect to time after addition of H_2_O_2_ (10 μM) with excitation at 490 nm.

In this work, we report a novel Aβ-targeted and blood–brain barrier (BBB)-permeable ratiometric H_2_O_2_-responsive fluorescent probe, R-MA-SLM (Figure 1A), based on a methylamino-substituted quinolinium-based cyanine fluorophore with a phenylboronate detection site for real-time imaging and monitoring of the H_2_O_2_ level induced by Aβ at the cellular level and in the brains of three different age groups of AD mice. Upon the addition of H_2_O_2_, the fluorescence intensity of R-MA-SLM progressively diminished at 574 nm concomitant with a gradual increase in the emission intensity at 661 nm when excited at 490 nm, providing dual-channel emissions and ratiometric monitoring. Intriguingly, R-MA-SLM showed a more than 4-fold faster response toward H_2_O_2_ in the presence of Aβ aggregates concomitant with an enhanced fluorescence intensity, offering an advantage for Aβ-induced H_2_O_2_ detection and monitoring. In addition to high stability and low cytotoxicity, this probe exhibited highly selective binding toward Aβ and good blood–brain barrier permeability, indicating it has tremendous potential for real-time detection and monitoring of Aβ-induced H_2_O_2_ in live cells and animal models. Indeed, the R-MA-SLM probe was able to detect and exhibit a ratiometric response toward endogenous H_2_O_2_ induced by Aβ in N2aSW cells. More importantly, real-time visualization and differentiation of different H_2_O_2_ contents induced by Aβ species in various age groups of 5XFAD Tg mice were successfully demonstrated by the probe, signifying it has potential for practical applications in the early diagnosis of AD as well as for understanding AD pathogenesis and progression and the evaluation of potential drugs during treatment.

## 2 Experimental Section

### 2.1 General Procedures

All of the solvents were dried by the standard methods wherever needed. ^1^H NMR spectra were recorded on a Bruker Avance-III HD 400 NMR spectrometer, and the NMR signals were referenced to the residue CHCl_3_ at 7.26 ppm or DMSO-*d*_6_ at 2.5 ppm. ^13^C NMR spectra were recorded on a Bruker Avance-III HD 400 NMR spectrometer, and the NMR signals were referenced to CDCl_3_ at 77 ppm or DMSO-*d*_6_ at 39.5 ppm. Mass spectroscopy (MS) measurements were carried out using fast atom bombardment on an API ASTER Pulser I Hybrid mass spectrometer or a matrix-assisted laser desorption ionization-time-of-flight (MALDI-TOF) mass spectrometer, Bruker Autoflex MALDI-TOF MS. For column chromatography, silica gel (230–400 mesh) with particle sizes of 38–63 μm was used, and for TLC, TLC Silica gel 60 F_254_ (25 aluminum sheets 20 × 20 cm) was employed. The saturated brine prepared from more than 36 g of sodium chloride in 100 mL of H_2_O was generally used as a quencher.

### 2.2 Synthesis of R-MA-SLM

Compounds **1** and **2** outlined in Scheme S1 were prepared by modified literature procedures.^24,25^

#### 4-(4,4,5,5-Tetramethyl-1,3,2-dioxaborolan-2-yl)benzyl(4-(hydroxymethyl)phenyl)(methyl) Carbamate (**3**)

To (4-(methylamino)phenyl)methanol (411 mg, 3 mmol) in THF (15 mL) cooled to 0 °C was added a saturated Na_2_CO_3_ solution (9 mL). Then, compound **1** (1.04 g, 3.5 mmol) in 30 mL of CH_2_Cl_2_ and 15 mL of a saturated Na_2_CO_3_ solution were added dropwise into the reaction mixture. After stirring for 2.5 h at room temperature, the reaction was quenched with water and then extracted thrice with ethyl acetate. The combined organic extract was washed with water and saturated brine. The organic layer was dried over anhydrous sodium sulfate, filtered, and then rotary evaporated. The residue was purified by column chromatography on silica gel (PE:EA = 4:1) to afford **3** (643 mg) as a yellow oil in 54% yield. ^1^H NMR (400 MHz, CDCl_3_, δ): 7.76 (d, *J* = 7.6 Hz, 2H), 7.34 (s, 2H), 7.32–7.26 (m, 3H), 7.22 (d, *J* = 7.6 Hz, 2H), 5.15 (s, 2H), 4.66 (s, 2H), 3.29 (s, 3H), 1.33 (s, 12H). ^13^C NMR (100 MHz, CDCl_3_, δ): 155.6, 139.7, 135.1, 127.6, 127.0, 84.0, 75.2, 67.4, 64.9, 25.0. HRMS (MALDI-TOF) *m*/*z* Calcd for C_22_H_28_BNO_5_: 398.2143. Found: 398.2130 [M + H]^+^.

#### 4-(4,4,5,5-Tetramethyl-1,3,2-dioxaborolan-2-yl)benzyl(4-formylphenyl)(methyl) Carbamate (**4**)

To the solution of compound **3** (500 mg, 1.26 mmol) in CHCl_3_ was added MnO_2_ (1.1 g, 12.6 mmol). The solution mixture was heated to reflux for 24 h. After cooling to room temperature, the reaction mixture was quenched with water and extracted thrice with ethyl acetate. The combined organic extract was washed with water and saturated brine. The organic layer was dried over anhydrous sodium sulfate and filtered. The solvent was then removed by rotary evaporation. The residue was purified by chromatography on silica gel (PE:EA = 3:1) to afford **4** (423 mg) as a pale yellow solid in 85% yield. ^1^H NMR (400 MHz, CDCl_3_, δ): 9.85 (s, 1H), 7.75 (d, *J* = 8.0 Hz, 2H), 7.71 (d, *J* = 8.0 Hz, 2H), 7.36 (d, *J* = 8.4 Hz, 2H), 7.24 (d, *J* = 7.6 Hz, 2H), 5.11 (s, 2H), 3.28 (s, 3H), 1.24 (s, 12H). ^13^C NMR (100 MHz, CDCl_3_, δ): 191.1, 154.8, 148.6, 139.1, 135.1, 133.3, 130.4, 127.2, 125.0, 83.9, 67.8, 37.2, 24.9. HRMS (MALDI-TOF) *m*/*z* Calcd for C_22_H_26_BNO_5_: 396.1986. Found: 396.1979 [M + H]^+^.

#### (*E*)-1-Methyl-4-(4-(methyl(((4-(4,4,5,5-tetramethyl-1,3,2-dioxaborolan-2-yl)benzyl)oxy)carbonyl)amino)styryl)quinolin-1-ium Iodide (R-MA-SLM)

A reaction mixture containing compound **4** (200 mg, 0.5 mmol), **2** (144 mg, 0.5 mmol), and piperidine (0.5 mL) in ethanol (15 mL) was heated at reflux overnight. After cooling to room temperature, the organic solvent was removed by rotary evaporation. The residue was first purified by silica gel column chromatography and followed by precipitation from methanol and ethyl acetate to yield R-MA-SLM (165 mg) in 50% yield. ^1^H NMR (400 MHz, CDCl_3_, δ): 9.83 (d, *J* = 6.0 Hz, 1H), 8.71 (d, *J* = 8.0 Hz, 1H), 8.29 (d, *J* = 6.4 Hz, 1H), 8.13–8.06 (m, 2H), 7.97–7.94 (m, 1H), 7.82–7.79 (m, 3H), 7.82–7.79 (m, 3H), 7.36 (t, *J* = 8.0 Hz, 4H), 5.23 (s, 2H), 4.60 (s, 3H), 3.36 (s, 3H), 1.25 (s, 12H). ^13^C NMR (100 MHz, CDCl_3_, δ): 155.2, 153.3, 148.8, 145.8, 142.8, 139.4, 138.8, 135.6, 135.2, 132.1, 130.0, 129.5, 127.4, 126.9, 125.7, 118.9, 118.8, 117.3, 84.1, 67.9, 45.9, 37.5, 25.0. HRMS (MALDI-TOF) *m*/*z* Calcd for C_33_H_26_BN_2_O_4_: 535.2768. Found: 535.2759 [M]^+^.

### 2.3 Aβ_1–40_ Fibrils Preparation

The Aβ_1–40_ fibrils were prepared according to our previously reported procedures^26^ as follows.

To prepare a stock solution of Aβ_1–40_, 1 mg of Aβ_1–40_ powder purchased from r-Peptide (GA, USA) without any purification was dissolved in 400 μL of 1% ammonium solution, which was then stored at −20 °C before use. The Aβ_1–40_ fibrils were prepared by incubating 50 μM Aβ_1–40_ monomer in 0.1 M phosphate buffer (20 mM, pH 7.4) at 37 °C for 24 h. The fibrils were then characterized by TEM to confirm its formation, and DLS was used to determine its size prior to use. (Figure S1)

### 2.4 In-Vitro Binding Assays

To determine the dissociation constant (*K*_d_) of the fluorophore, a thioflavin T (ThT) displacement assay by R-MA-SLM and MA-SLM was adopted. The fluorescence intensity of the ThT/Aβ_1–40_ fibril complex prepared in advance was monitored with an excitation at 440 nm. Then, various concentrations of R-MA-SLM or MA-SLM solution (2–14 μM, 7.5 μL) were added to the ThT/Aβ_1–40_ fibril complex (100 μM, 7.5 μL) in phosphate buffer (25 mM, pH = 7.4). Before performing measurements, the mixture was incubated at room temperature for 5 min. The emission intensity of the two probes was measured under excitation of the respective maximum absorption wavelength. When the dye concentration was increased, the emission intensity of ThT at 480 nm decreased accordingly; meanwhile, the fluorescence intensity at 565/660 nm of R-MA-SLM/MA-SLM increased. Emission spectra of the mixture were measured by a HORIBA FluoroMax-4 Spectro Fluorometer. The dissociation constant was then determined by fitting into the corresponding saturation binding curves.

### 2.5 Cell Viability Assays

The cytotoxicity of R-MA-SLM and MA-SLM on SH-SY5Y cells was investigated by classical 3-(4,5-dimethyl-2-thiazolyl)-2,5-diphenyl-2*H*-tetrazolium bromide (MTT) assay. The previously employed literature procedure was adopted.^26^

### 2.6 Cell Imaging Experiments

N2a mouse neuroblastoma cells stably expressing Swedish mutant APP, which were a generous gift from Dr. Gopal Dinakaran (University of Chicago, Chicago, IL, USA), were employed in the imaging experiments. N2aSW cells are N2a cells expressing human APP Swedish mutations that secrete high levels of Aβ peptides and beta-secretase-generated soluble APP derivatives (APPs beta). For colocalization studies, N2aSW cells were plated in a sterilized coverslip in the 24-well plate and cultured in the wells with 7000 cells/mL for 24 h. The cells were first incubated with diphenyleneiodonium iodide (DPI) inhibitor for 15 min. After that, the cells were treated with R-MA-SLM and MA-SLM for 0.5 h. Then, cells were fixed with 4% PFA for 15 min and washed with PBS buffer twice. Subsequently, the cells were permeabilized with 0.1% PBST for 15 min and blocked with 1% BSA buffer for 1 h. To costain with dye or antibody, the cells were incubated with Thio-S for 3 min or 6E10, A11, 4G8 primary antibody for 2 h. After removal of Thio-S or primary antibody, the cells were washed with PBS buffer twice. For antibody labeling, the cells were incubated with the secondary antibody for 1 h. Lastly, the cells were washed with PBS buffer twice and then stained with DAPI for 4 min followed by washing with PBS buffer. After that, the coverslip was mounted in the glass slide and observed using confocal microscopy.

For H_2_O_2_ imaging studies, N2a and N2aSW cells were seeded in a glass-bottomed culture dish at a concentration of 3 × 10^4^ cells/well and cultured for 24 h in DMEM in an incubator (37 °C, 5% CO_2_). For exogenous H_2_O_2_ detection, after being treated with diphenyleneiodonium iodide (DPI) (2 μM) for 15 min, N2a and N2aSW cells were incubated with R-MA-SLM for 5 min and then further added with H_2_O_2_ (20 μM). Cells were washed three times with PBS (pH 7.4) buffer prior to imaging. The images were recorded from the green channel (λ_em_ = 540–600 nm) and the red channel (λ_em_ > 650 nm) with excitation at 488 nm at different time points. For endogenous H_2_O_2_ detection, N2a and N2aSW cells were adhered to the culture dish for 24 h in an incubator (37 °C, 5% CO_2_). After being incubated with R-MA-SLM (10 μM) for 5 min, N2a and N2aSW cells were kept at different time points. Before imaging, the cells were washed three times with PBS (pH 7.4) buffer. Fluorescence images were acquired from the green channel (λ_em_ = 540–600 nm) and the red channel (λ_em_ > 650 nm) with excitation at 490 nm.

To determine the effect of R-MA-SLM on non-neuronal cells, immortalized microglial (IMG) cells were used and treated with R-MA-SLM (10 μM) at various time-points (0, 5, 10, 15, and 30 min). Cells were then washed once with PBS (1×) followed by fixation with PFA (4%) for 15 min. The fixed cells were washed 3 times with PBS, counter-stained with nuclear stain (DAPI) for 5 min, and mounted onto clean glass slides. Fluorescence cells images visualized by a confocal microscope (Leica Microsystems) were acquired from the green channel (λ_em_ = 540–600 nm) and the red channel (λ_em_ > 650 nm) with excitation at 490 nm.

### 2.7 Animal

Before performing all animal experiments, approval from the Committee on the Use of Human and Animal Subjects in Teaching and Research (HASC) of Hong Kong Baptist University was obtained. (HASC#20-21). Valid and relevant licenses under the Animals Ordinance were also obtained from the Department of Health, Hong Kong under the animal license nos. 17–77 in DH/SHS/8/2/6 Pt.1 and 20–26 in DH/HT&A/8/2/6 Pt.1. All methods adopted were performed according to the approved guidelines and regulations in all experiments. The 5XFAD transgenic mice used in this study were purchased from the Jackson Laboratory (Bar Harbor, ME, USA) and housed in the animal unit of the School of Chinese Medicine of HKBU.

### 2.8 In-Vivo Near-Infrared Fluorescence Imaging of R-MA-SLM

The previously adopted imaging procedures^13,28^ were followed in which 5XFAD transgenic mice (2, 6, and 12 month old) and age-matched wild-type mice were studied by tail-vein injection of 100 μL of R-MA-SLM (20 mg/kg).

### 2.9 Ex-Vivo Costaining of Brain Slices of 5XFAD Mice

The previously adopted costaining procedures^27,28^ were followed in which 5XFAD mice were first injected intravenously with R-MA-SLM followed by costaining the brain sections with either thioflavin-S (Thio-S) fluorescence dye or primary antibody such as 4G8, 6E10, or A11 before performing the confocal imaging.

## 3 Results and Discussion

### 3.1 Synthesis and Photophysical Properties of the Probe

The synthesis of the R-MA-SLM fluorophore is outlined in Scheme S1. The Knoevenagel condensation of boronate-substituted benzaldehyde and dimethylquinolinium salt in the presence of piperidine was adopted as a key step to synthesize the desired H_2_O_2_-responsive fluorophore R-MA-SLM, which was fully characterized by ^1^H and ^13^C NMR spectroscopy as well as high-resolution mass spectrometry. The acquired data fully agree with the proposed structure.

R-MA-SLM has a broad absorption peak at 388 nm and a relatively weak emission band at 574 nm upon excitation at the absorption maximum in PB with a fluorescence quantum yield (Φ_PL_) of 2.7%. When H_2_O_2_ was added, the absorption and emission gradually red shifted to 485 and 661 nm, respectively, concomitant with a progressive increase in intensity over time. (Figure S2, Figure 1B, and Table S1) Such changes were due to the oxidative cleavage of the boronate ester of R-MA-SLM by H_2_O_2_, yielding a new NIR fluorophore MA-SLM (Figure 1A). The strong donor–acceptor intramolecular charge transfer of MA-SLM gave rise to the red shift of the absorption and emission bands. The dual-channel emissive characteristic of R-MA-SLM offers a ratiometric fluorescence sensing capability for H_2_O_2_ detection and monitoring.

Intriguingly, the R-MA-SLM probe exhibited not only strong fluorescence enhancement but also a faster (>5-fold) response to H_2_O_2_ in the presence of Aβ_1–40_ fibrils in which the Aβ_1–40_ peptide is the predominant form in the brain. (Figure 1B) The large increase in fluorescence was likely due to the strong binding interactions between the probe (and its oxidized product) and the Aβ_1–40_ fibril, which reduced its nonradiative decay. To investigate the binding affinity of R-MA-SLM and MA-SLM with Aβ, a displacement assay of thioflavin T (ThT)-bound Aβ_1–40_ fibrils with R-MA-SLM and MA-SLM was performed (Figures S3 and S4), in which the dissociation constants (*K*_d_) of R-MA-SLM and MA-SLM with Aβ_1–40_ fibrils were determined to be 3.80 and 0.58 μM, respectively, indicating the strong binding affinity between them (Table S2). The kinetic characteristics of the probe in response to H_2_O_2_ in the absence and presence of Aβ were also estimated from the time-dependent fluorescence changes of R-MA-SLM. The observed reaction rate constant (*k*_obs_) of the pseudo-first-order reaction in the absence of Aβ_1–40_ (2.4 × 10^–4^ s^–1^) was found to be smaller than that (3.0 × 10^–3^ s^–1^) in the presence of Aβ_1–40_ under the reaction conditions shown in Figures S5 and S6. In addition, the fluorescence intensity *F*_660_/*F*_574_ ratio exhibited excellent linearity against the concentration of H_2_O_2_ in the range of 2–10 μM with a linear coefficient of 0.9960 in the presence of Aβ_1–40,_ indicating that R-MA-SLM can be applied for quantitative determination of the H_2_O_2_ content within this range. The limit of detection (LOD) was thus estimated to be 0.17 μM, which was significantly lower than that without Aβ (LOD = 0.26 μM) (Figures S7 and S8). These results affirmed that R-MA-SLM could exhibit a rapid and highly sensitive response to H_2_O_2_ in the presence of Aβ, offering a remarkable ability to ratiometrically detect and monitor endogenous H_2_O_2_ induced by Aβ on a real-time scale.

### 3.2 Sensing Mechanism of the Probe toward H_2_O_2_

The phenylboronate functionality of the probe is vulnerable to attack by H_2_O_2_ at the electrophilic boron center, first forming a tetrahedral boronate intermediate. The aryl group then undergoes 1,2-migration from the boron atom to the adjacent oxygen atom, yielding borate. Subsequently, borate hydrolyzes readily under physiological conditions, resulting in a boric acid/ester and the corresponding phenolic intermediate. Last, the methylphenyloxy linker in the phenolic intermediate cleaves spontaneously through 1,6-elimination, yielding the oxidized product MA-SLM.

To confirm the proposed detection mechanism of R-MA-SLM, high-performance liquid chromatography-high-resolution mass spectrometry (HPLC-HRMS) was employed to analyze the product formed from reaction of the probe and H_2_O_2_. As clearly shown in the HPLC trace and HRMS spectrum for the reaction of R-MA-SLM and H_2_O_2_ (Figure S9), the peak was observed with a retention time (*t*_R_) at 3.909 min, showing a base peak at *m*/*z* 275.1561 in the mass spectrum corresponding to MA-SLM. As a result, the proposed detection mechanism was unambiguously demonstrated and confirmed.

### 3.3 Stability, Selectivity, and pH Effect of the Probe

The fluorescence intensity *F*_660_/*F*_574_ ratio of R-MA-SLM in the absence and presence of H_2_O_2_ after complete reaction at approximately 60 min and saturation and the fluorescence intensity of the R-MA-SLM under ambient light illumination over a period of 90 min at ambient temperature measured at the emission maximum showed no significant variation (Figure S10), indicating good photostability of the probe under the working conditions. Furthermore, it was found that the R-MA-SLM probe exhibited high selectivity and specificity toward H_2_O_2_ over other ROS, reactive nitrogen species (RNS), metal ions, and bioactive small molecules without and with Aβ_1–40_ fibrils. When different types of interference reagents were added to the R-MA-SLM solution, the change in the ratio of the fluorescence intensity of *F*_660_/*F*_574_ was shown to be insignificant for all of the tested reagents (Figure S11). In addition, the effect of pH on the fluorescence intensity of the probe in the presence and absence of H_2_O_2_ was studied. The fluorescence intensity *F*_660_/*F*_574_ ratio exhibited no substantial change for the free probe and the probe after the addition of H_2_O_2_ over a pH range of 6.0–8.0 (Figure S12). All of these desirable properties are indispensable for probes in biosensing applications.

### 3.4 Cytotoxicity and Detection of H_2_O_2_ at the Cellular Level

Before being applied for biological studies, the cytotoxicity of R-MA-SLM and MA-SLM against human neuroblastoma (SH-SY5Y) cells using the 3-(4,5-dimethyl-2-thiazolyl)-2,5-diphenyl-2*H*-tetrazolium bromide (MTT) viability assay was carried out. As shown in Figure S13, R-MA-SLM and MA-SLM showed low cytotoxicity over a wide concentration range with LC_50_ values of >200 and 46.6 μM, respectively, suggesting that they are biocompatible for bioapplications.

To evaluate the targeting ability of the probe, R-MA-SLM, and its oxidized product MA-SLM toward various Aβ species in live N2aSW cells, which are N2a mouse neuroblastoma cells expressing human APP Swedish mutations, we conducted colocalization studies with the Aβ-fibril staining fluorescent dye thioflavin-S (Thio-S) and various Aβ antibodies including Aβ oligomer (A11) antibody, anti-Aβ antibodies, 6E10, and 4G8. As shown in Figure S14, the images of R-MA-SLM and MA-SLM merge well with the images of Thio-S and various Aβ antibodies with the average Pearson’s colocalization coefficient (*R*) in the range of 0.88–0.99. These results have clearly demonstrated that the probe R-MA-SLM and its oxidized product MA-SLM showed excellent Aβ targetability in living cells.

**Figure 2:**
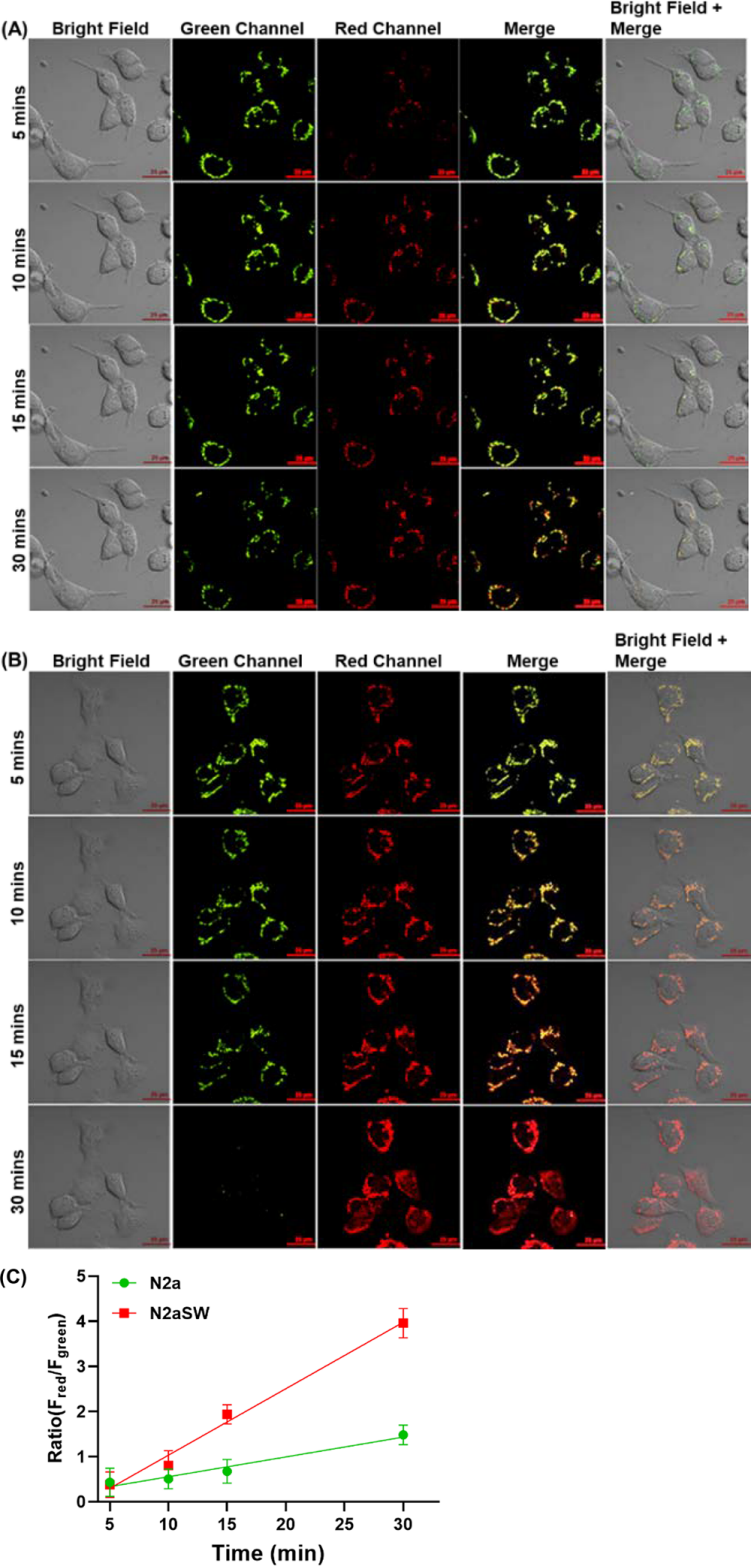
Confocal fluorescence images of (A) N2a and (B) N2aSW cells at different time points after being incubated with R-MA-SLM for 5 min. Fluorescence images were acquired from the green channel (λ_em_ = 540–600 nm) and red channel (λ_em_ > 650 nm) with excitation at 490 nm. Scale bar: 25 μm. (C) Plots of the corresponding fluorescence intensity ratio (*F*_red_/*F*_green_) of N2a and N2aSW cells at different time points. Data are expressed as the mean ± SD of three independent measurements (*n* = 3).

To evaluate the capability of R-MA-SLM as a ratiometric fluorescent probe to directly detect and image H_2_O_2_ induced by Aβ at the cellular level, imaging studies of endogenous H_2_O_2_ in neuronal cells, N2a and N2aSW cells, and non-neuronal cells, immortalized microglial (IMG) cells were carried out. The R-MA-SLM probe was found to have fast uptake by N2a cells, providing fluorescence in dual-emission channels: a green channel (λ_em_ = 500–600 nm) and a red channel (λ_em_ = 650–700 nm) upon excitation at λ_ex_ = 490 nm. After pretreatment with diphenyleneiodonium iodide (DPI), an H_2_O_2_ inhibitor, R-MA-SLM-loaded N2a and N2aSW cells showed fluorescence only in the green channel (Figure S15). However, upon addition of H_2_O_2_ to these DPI-pretreated N2a cells, the fluorescence in the green channel gradually faded out whereas the fluorescence in the red channel progressively became more intense over time, confirming the capability of R-MA-SLM for ratiometric fluorescence detection of H_2_O_2_ in living cells. Furthermore, the ability of the probe to respond and track endogenous H_2_O_2_ induced by Aβ in N2a and N2aSW cells as well as non-neuronal IMG cells was investigated. Figure 2 shows the fluorescence images of N2aSW cells in the green and red channels at different time points after incubation with R-MA-SLM for 5 min. It is clearly shown that the green fluorescence gradually diminishes and the red fluorescence becomes brighter over time. It is worth mentioning that the ratiometric color change occurs at a much faster rate accompanied by stronger fluorescence intensities in N2aSW cells than in N2a cells (Figure 2C), in which a larger change in response of the probe as a function of concentration of H_2_O_2_ was evidenced. It is attributed to the Aβ-enhanced reaction kinetics of the probe responsive to endogenous H_2_O_2_ induced by Aβ as well as the Aβ-binding-induced fluorescence enhancement of the probe and its oxidized product MA-SLM. In sharp contrast, the scattered and weak green fluorescence shows no obvious decrease in intensity, and there is also no red fluorescence being observed over time in IMG cells (Figure S16), indicating that IMG cells show no overproduction of H_2_O_2_ in the absence of Aβ. These results strongly suggest that the new R-MA-SLM probe is a highly sensitive and fast responsive tool to ratiometrically detect and monitor endogenous H_2_O_2_ in living cells and to differentiate AD cells from their normal counterparts, highlighting its great potential for H_2_O_2_ detection and monitoring in an AD mouse model in vivo.

### 3.5 In-Vivo Imaging and Monitoring of H_2_O_2_ in a Mouse Model

The ability of a probe to cross the blood–brain barrier is indispensable for in-vivo real-time brain imaging. Hydrophobic molecules with an index of lipophilicity (log *P*) of 2.0–3.5 have a high propensity to cross the BBB. The log *P* value of R-MA-SLM was first estimated to be 2.76 using the online ALOGPS 2.1 program. To experimentally investigate the BBB penetrability of R-MA-SLM, a biodistribution study of R-MA-SLM in different organs over time after intravenous injection of the probe in 2-month old wild-type (WT) and 5XFAD (2, 6, and 12-month old) mice was performed. As seen in Figure S17, fluorescence signals were primarily observed in the liver, kidney, and brain 6 h postinjection in the green channel, showing that R-MA-SLM was BBB permeable. Forty-eight hours postinjection, the fluorescence intensities decreased significantly among all of these organs. There were almost no fluorescence signals seen in these organs at 72 h postinjection, indicating that R-MA-SLM could be metabolized and excreted from the body of the tested mouse. Consistently, the fluorescence spectra and mass spectroscopic studies by ESI-MS of urine samples collected from 2-month old WT and 5XFAD mice before and after administration of R-MA-SLM at different time points showed that the probe and its reaction products were fast eliminated through urine excretion over time (Figure S17B and S17C). Furthermore, the brain extract of the R-MA-SLM-treated WT mouse at 6 h postinjection was analyzed by ESI-MS in which the molecular ion (M^+^) peak with *m*/*z* = 535.2 was found in the mass spectrum (Figure S18). This result further confirms that R-MA-SLM can cross the BBB intact. In addition, the toxicity of R-MA-SLM in mice was investigated. As shown in Figure S19, there were no obvious changes in the body weight of the R-MA-SLM-treated WT and 5XFAD (2, 6, and 12 month old) mice at different time points. Furthermore, the weight of the collected organs from R-MA-SLM-treated 5XFAD mice did not show any noticeable weight changes relative to those of the untreated WT mouse. The H&E staining of the different organs in the treated 5XFAD mice also did not exhibit any histopathological changes as compared to the untreated WT one. Lastly, blood parameter analysis did not demonstrate any evident changes in the biochemical constituents of blood in the treated 5XFAD relative to that of the WT control. In short, these results fully supported that this ROS probe is nontoxic and highly biocompatible.

**Figure 3:**
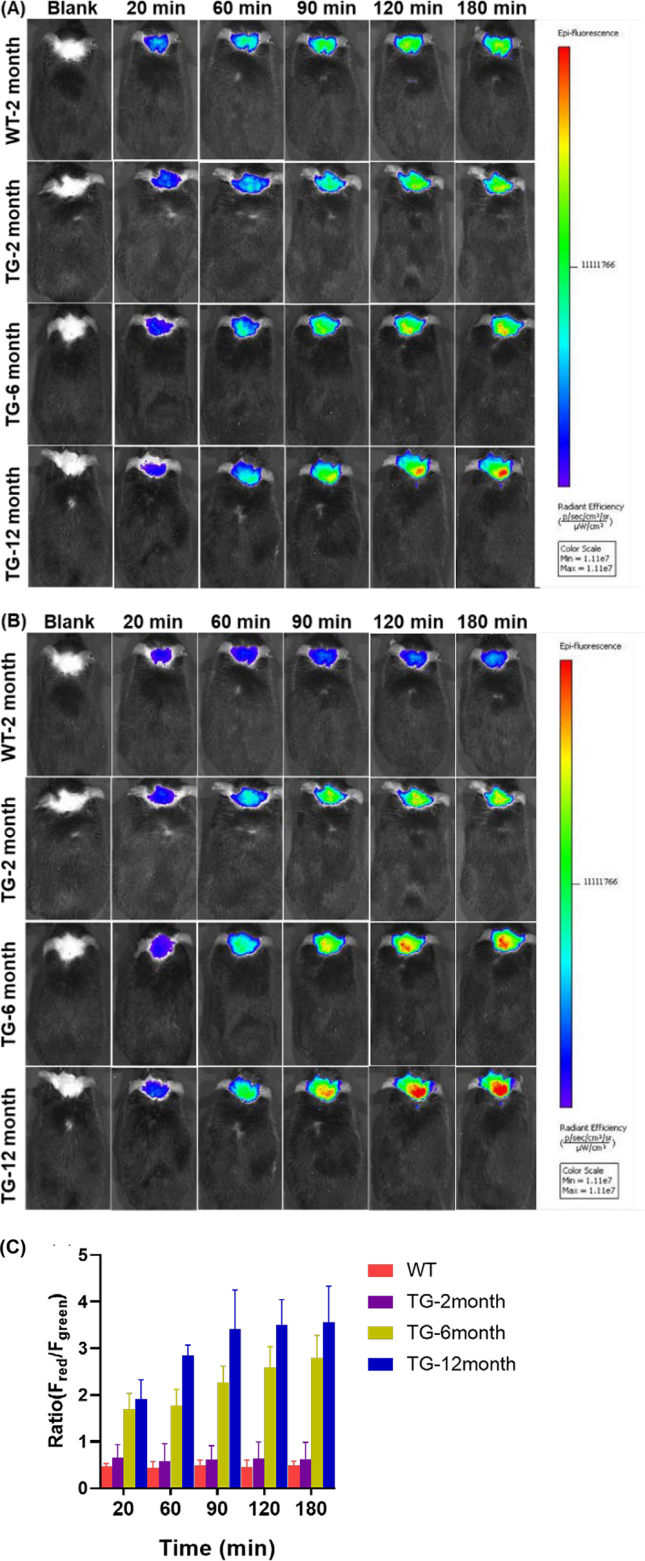
In-vivo dual-channel fluorescence images of 2-month old wild-type and 5XFAD (12, 6, and 2-month old) mice at different time points before and after IV injection of R-MA-SLM (20 mg/kg) in 100% PEG-600 acquired from (A) the green channel (λ_em_ = 575–650 nm) and (B) the red channel (λ_em_ = 695–770 nm) with an excitation wavelength λ_ex_ of 500 nm. (C) Corresponding fluorescence intensity ratio *F*_red_/*F*_green_ of WT and 5XFAD mice at different ages monitored over a period from 20 to 180 min. Data are expressed as the mean ± SD of three independent mice (*n* = 3).

To assess the H_2_O_2_-responsive capability of R-MA-SLM in the brains of AD mouse models, in-vivo cerebral fluorescence intensity comparison among different age groups, i.e., 2, 6, and 12-month old, 5XFAD mice that overexpressed Aβ species and their 2-month old WT counterparts at different time points was used. As shown in Figure 3, the fluorescence signals in the brain of the 2-month old WT mouse in both the green (λ_em_ = 575–650 nm) and the red (λ_em_ = 695–770 nm) channels increased over time, further affirming the BBB permeability of the probe, whereas the corresponding fluorescence intensity ratio (*F*_red_/*F*_green_) of the dual channels remained fairly constant at all time points, indicating no obvious alteration of the H_2_O_2_ level in the brain of the WT mouse over a period of 3 h. In contrast_,_ the brains of the 2-month old 5XFAD mice exhibited significantly higher fluorescence signals than those of the age-matched WT mouse in both of the emission channels at all time points, whereas the corresponding fluorescence intensity ratios (*F*_red_/*F*_green_) were at a similar level as compared to those of the WT, implying that there was insignificant H_2_O_2_ production in the brain of the 2-month old 5XFAD mice. As cerebral Aβ aggregates were barely built up at the age of 2 months, the stronger fluorescence signals found in the brains of age-matched 5XFAD mice could mainly be attributed to the dysfunction of the BBB of Tg mice, leading to a higher concentration of the probe. Interestingly, the fluorescence signals in the dual channels and the corresponding fluorescence intensity ratio (*F*_red_/*F*_green_ increased from 0.5 to 3.3) showed an age-dependent increase among 5XFAD mice at all time points. The stronger fluorescence signals observed in the green channel in older AD mice are largely due to the strong Aβ-binding-induced fluorescence enhancement of the probe in which Aβ was shown to be more abundant in older AD mice. On the other hand, the age-dependent increase in fluorescence in the red channel giving rise to an increase in the *F*_red_/*F*_green_ ratio from 0.5 in 2-month old to 3.3 in 12-month old mice was attributed to the higher H_2_O_2_ level generated by Aβ in older AD mice. The above results clearly proved that cerebral Aβ species could induce significant H_2_O_2_ generation and thus could serve as an alternative biomarker for disease diagnosis and treatment. Furthermore, it was unambiguously demonstrated that the ratiometric R-MA-SLM probe was highly sensitive and responsive to visualize and differentiate different H_2_O_2_ levels induced by Aβ species in vivo in different age groups of transgenic AD mice.

**Figure 4:**
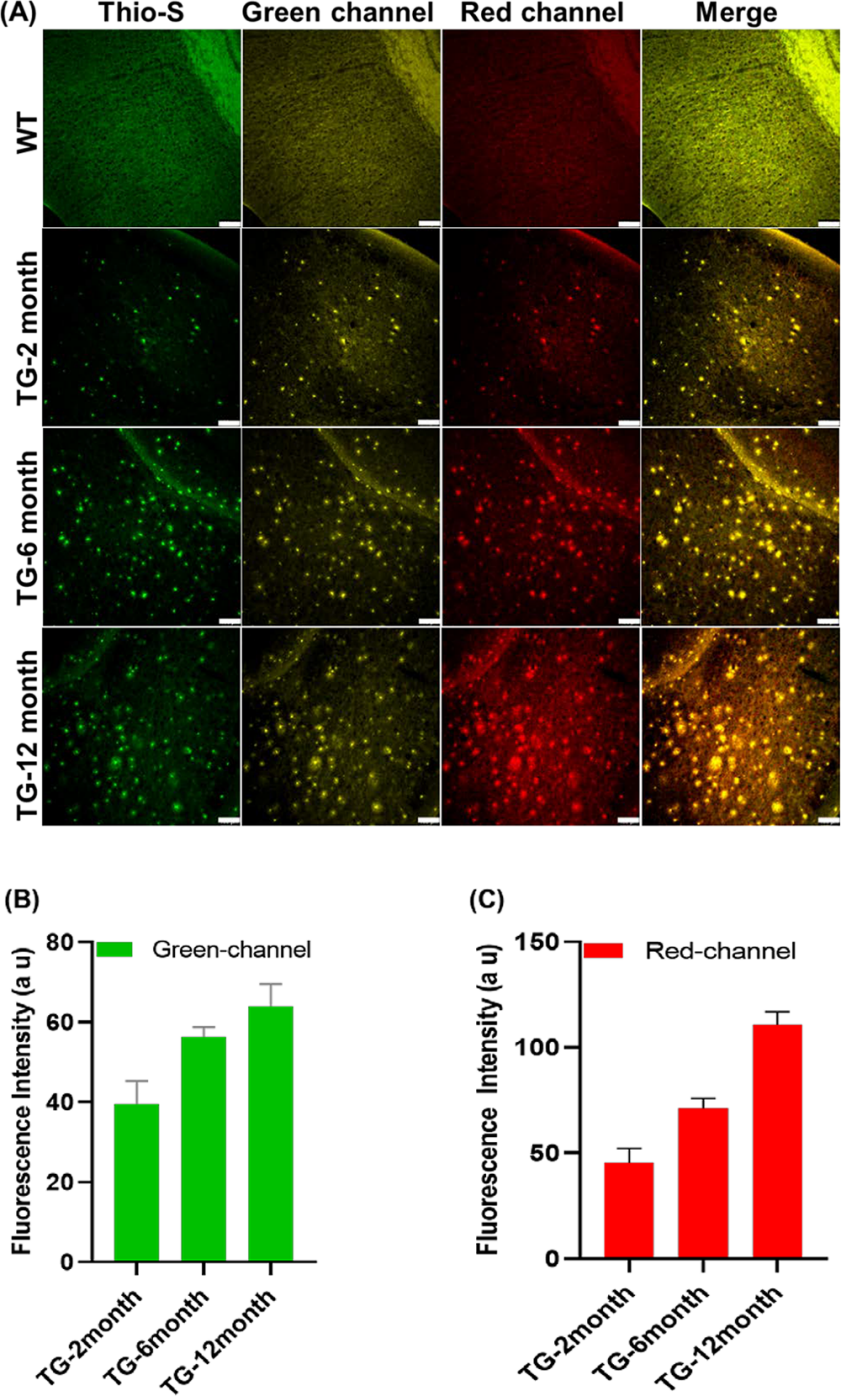
(A) Ex-vivo fluorescence images of brain slices of the WT mouse (2-month old) and 5XFAD mice at different age groups (2, 6, and 12-month old) postinjection of R-MA-SLM (20 mg/kg) for 60 min. Images were obtained from the green channel, λ_em_ = 540–600 nm, and the red channel, λ_em_ = 650–700 nm, with excitation at λ_ex_ = 488 nm. Scale bar: 100 μm. (B) Plot of the fluorescence intensity from the green channel against different age groups of Tg mice. Data are expressed as the mean ± SD of three independent measurements (*n* = 3). (C) Plot of the fluorescence intensity from the red channel against different age groups of Tg mice. Data are expressed as the mean ± SD of three independent measurements (*n* = 3).

Ex-vivo imaging of the brain slices of 5XFAD mice of different age groups and WT mice after postinjection of R-MA-SLM was also carried out to confirm the targeting capability of the probe and its oxidized product to various Aβ species in which various antibodies, including Aβ oligomer (A11) antibody, anti-Aβ antibodies, 6E10 or 4G8, and the Aβ-fibril specific labeling dye, Thio-S, were used for colocalization studies. Essentially, no fluorescent clusters were seen in various costained brain slices of the WT mouse (Figure 4 and Figure S20), suggesting that no Aβ species developed in the 2-month old WT mouse. It was distinctly shown that the fluorescent clusters that appeared in the brain slices of 5XFAD mice were highly age dependent in which the older Tg mice showed an increasing number of Aβ species in their brains. In light of the excellent overlap of the costained fluorescence images, the R-MA-SLM and MA-SLM probes demonstrated excellent targetability to various Aβ species in the brains of 5XFAD mice in different age groups, as shown in Figure 4 and Figure S20. Furthermore, the red fluorescence clusters emitted by MA-SLM, which are H_2_O_2_-oxidized products of R-MA-SLM, showed age-dependent increases in the size and quantity, suggesting a higher H_2_O_2_ level induced by a higher Aβ content in older AD mice. All of these findings are in good agreement with the results observed in the in-vivo imaging studies.

## 4 Conclusions

In summary, the first Aβ-targeted ratiometric H_2_O_2_-responsive fluorescent probe R-MA-SLM was developed for real-time detection and monitoring of changes in Aβ-induced H_2_O_2_ content in live cells and the brains of AD mouse models. Because of a large emission shift upon reacting with H_2_O_2_, enhanced response, high sensitivity and specificity, strong binding affinity with Aβ and Aβ-binding induced fluorescence enhancement, insignificant cytotoxicity, as well as excellent BBB penetrability and Aβ targetability, the R-MA-SLM probe was successfully applied for imaging real-time changes in H_2_O_2_ content induced by Aβ species in neuronal cells and a Tg AD mouse model. Such a highly sensitive probe was also able to differentiate different H_2_O_2_ levels induced by Aβ species in vivo in different age groups of Tg AD mice in which the cerebral H_2_O_2_ level increased age dependently concomitant with the plaque contents. The successful applications of the probe in vivo highlighted its versatility and promise as a powerful tool for facilitating an investigation of oxidative stress in the AD brain, a better understanding of the role of ROS in AD pathology, an early diagnosis of high-risk subjects, and the development of disease-modifying drugs for AD treatment.

## Supporting Information Available

Synthetic scheme, proposed sensing mechanism, fluorescence spectra, results of ThT fluorescence assay, results of kinetic studies, results of HPLC and HRMS analyses, stability results, selectivity plot, results of the pH effect, results of cell viability, colocalization analyses, confocal fluorescence images, results of biodistribution of the probe, ESI-MS spectrum of the brain extract, toxicity study, and ex-vivo fluorescence images (PDF)
