# Supplementary material for: Real-Time Detection and Visualization of Amyloid-β Aggregates Induced by Hydrogen Peroxide in Cell and Mouse Models of Alzheimer’s Disease
Source: ACS Appl Mater Interfaces. 2022 Jul 22;15(1):39–47. doi: 10.1021/acsami.2c07859 (PMC9837777; doi:10.1021/acsami.2c07859)
Supplement: Supplementary file 1 — am2c07859_si_001.pdf [file am2c07859_si_001.pdf]

Real-time Detection and Visualization of Amyloid-β Aggregates Induced Hydrogen Peroxide in Cell and Mouse Models of Alzheimer's Disease

Xueli Wang,[1,†] Ashok Iyaswamy,[2,†] Di Xu,[1] Senthilkumar Krishnamoorthi,[2,4] Sravan Gopalkrishnashetty Sreenivasmurthy,[2] Yuncong Yang,[1] Yinhui Li,[1] Chen Chen,[1] Min Li,[2] Hung-Wing Li,[3] and Man Shing Wong*,[1]*

1. Department of Chemistry, Hong Kong Baptist University, 224 Waterloo Road, Kowloon Tong, Hong Kong, SAR China. E-mail: mswong@hkbu.edu.hk

2. Mr. & Mrs. Ko Chi-Ming Centre for Parkinson's Disease Research, School of Chinese Medicine, Hong Kong Baptist University, 7 Baptist University Road, Kowloon Tong, Hong Kong, SAR China.

3. Department of Chemistry, The Chinese University of Hong Kong, Room 243, Science Centre, North Block, Shatin, Hong Kong, SAR China. E-mail: hungwingli@cuhk.edu.hk

4. Centre for Trans-disciplinary Research, Department of Pharmacology, Saveetha Dental College and Hospitals, 162, Poonamallee High Road, Chennai, Tamil Nadu 600077, India.

[†]X.W. and A.I. contributed equally to this work.

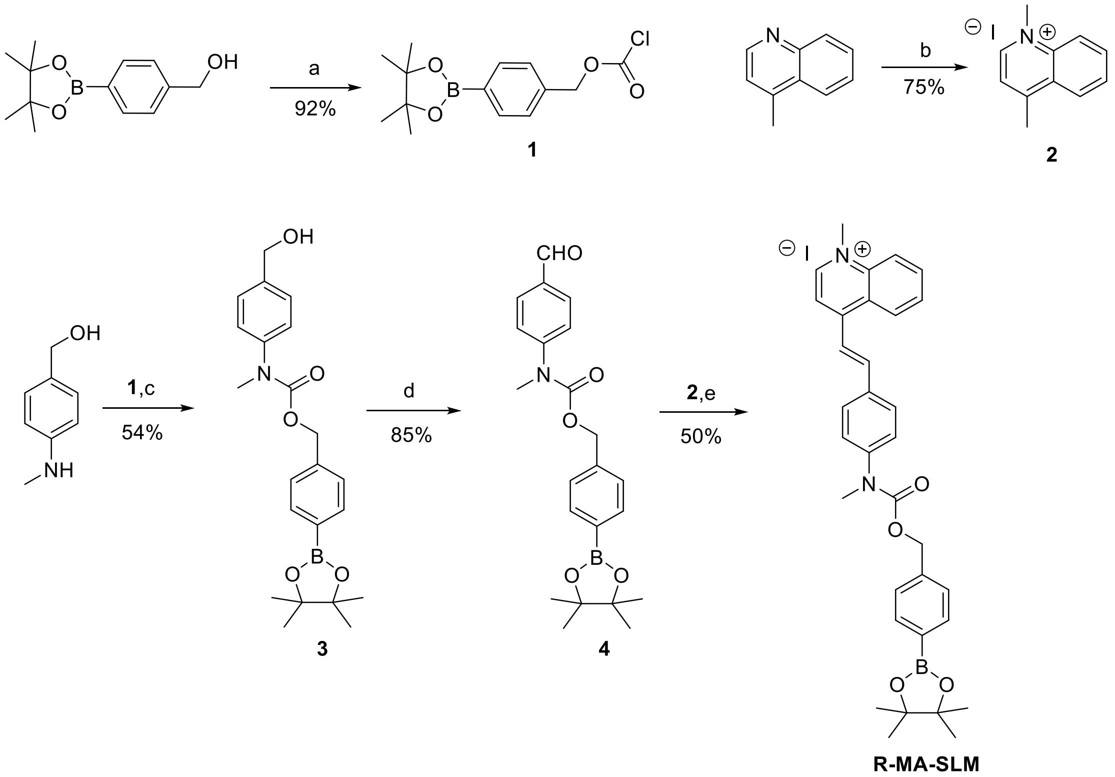

Reagents and Conditions: a) Na_2CO_3, triphosgene, toluene; b) CH_3I, MeCN, reflux; c) Na_2CO_3, THF/H_2O; d) MnO_2, $CHCl_3$; e) piperidine EtOH, reflux.

Scheme S1. The synthetic route of ratiometric fluorescence probe, **R-MA-SLM**.

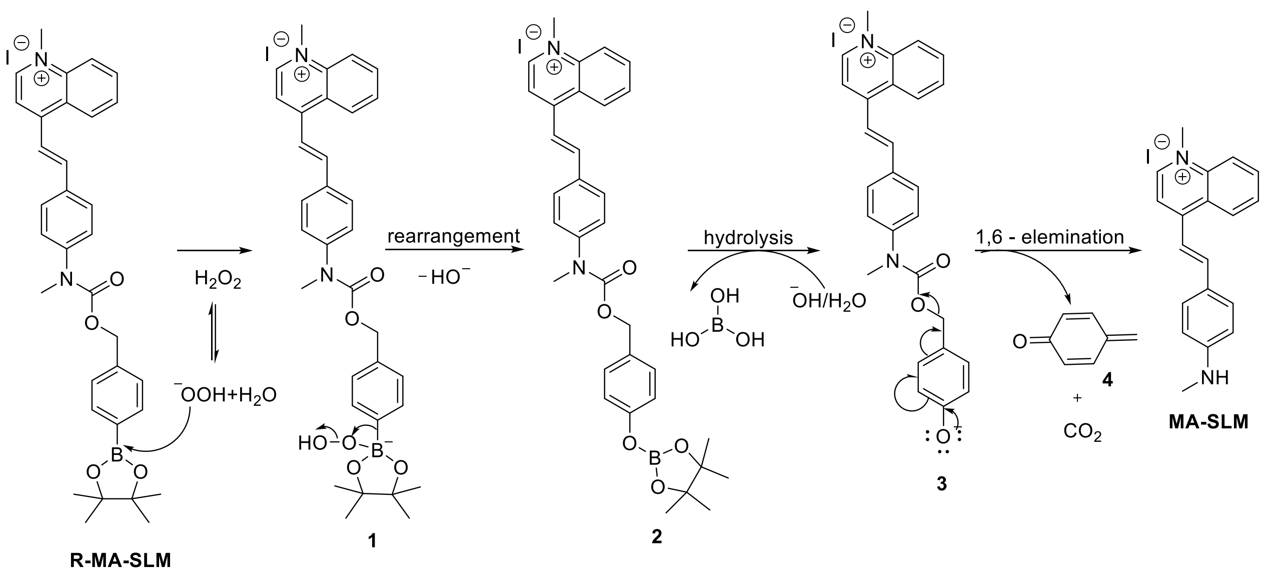

Scheme S2. Proposed mechanism of **R-MA-SLM** with H_2O_2.

(A)

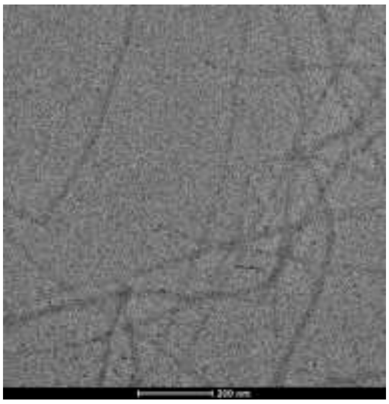

(B)

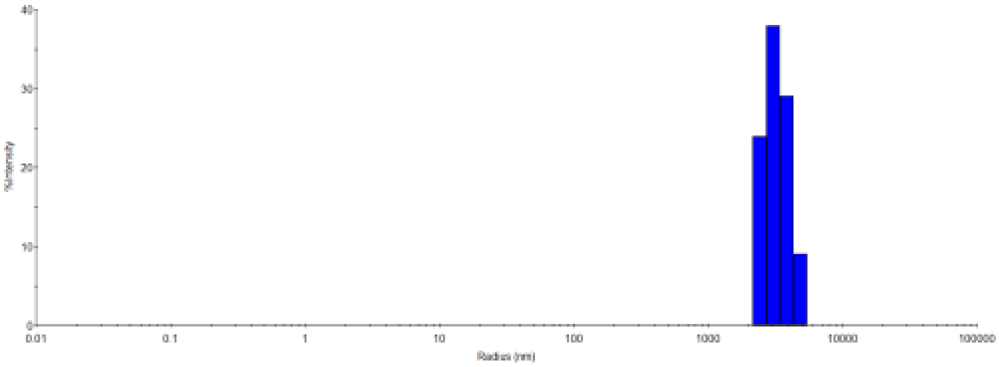

(C)

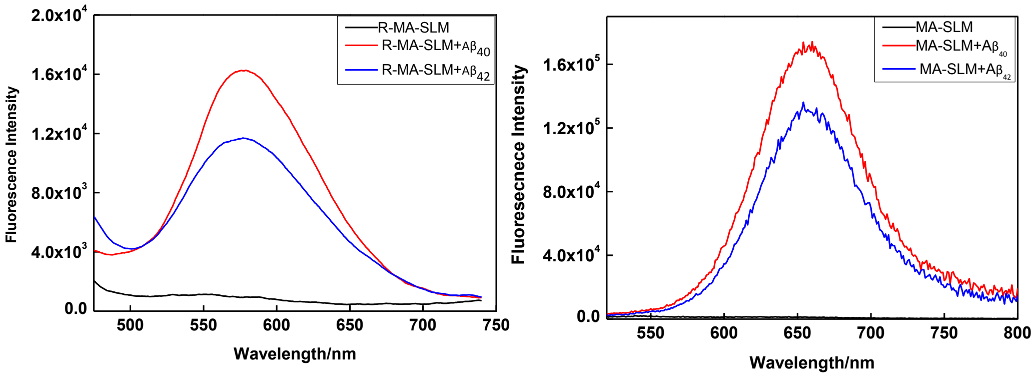

Figure S1. (A) Representative TEM image of Aβ_{1-40} fibrils. (B) Size distributions of Aβ_{1-40} fibrils (15 mM) in 50 mM PB buffer (pH = 7.4) measured by DLS. (C) Fluorescence spectra of **R-MA-SLM** (left) and **MA-SLM** (right) (20 μM) in the presence of 200 μM Aβ_{1-40} and Aβ_{1-42} fibrils, respectively.

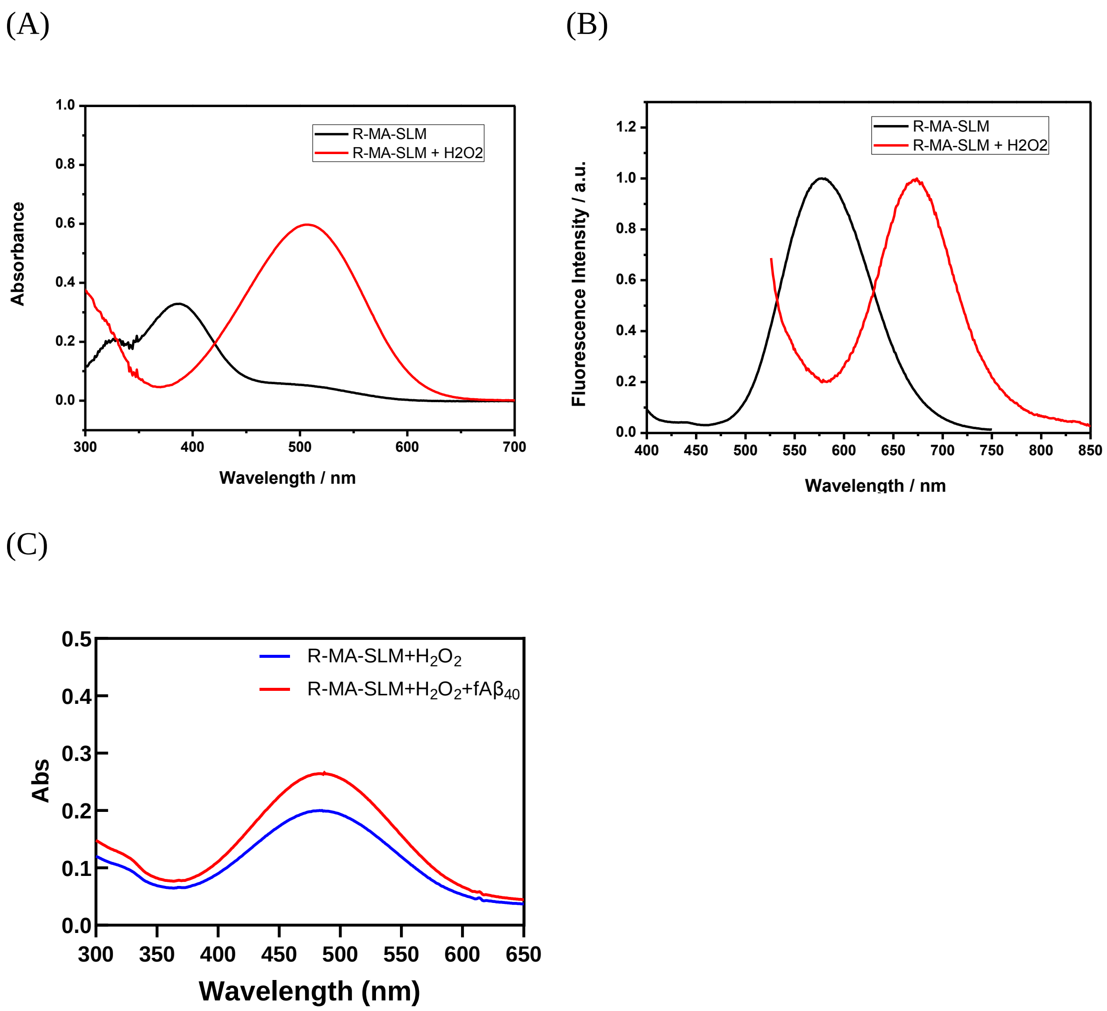

Figure S2. (A) Absorption and (B) Emission spectra of **R-MA-SLM** (50 μM) in the presence and absence of 50 mM H_2O_2 in PBS. (C) The absorption spectra of R-MA-SLM (50 μM) with 50 mM of H_2O_2 in the absence and presence of $A\beta_{1-40}$ fibril (250 μM).

Table S1. Summary of the optical properties of **R-MA-SLM** and **MA-SLM**.

Compound	Solvent	λ^{abs}_{max} [b] (ε)	λ^{em}_{max} [c] (Stoke Shift)	Φ (%)
**R-MA-SLM**	PB	388 (0.53)	574 (186)	2.7±0.3[d]
**MA-SLM**	PB	485 (1.68)	661 (176)	0.3±0.03[e]

PB is 0.1 M phosphate buffer solution; [b]Linear absorption maximum peak in the nm unit; ε is the molar absorptivity (10^4 $M^{-1}cm^{-1}$). [c]Fluorescence maximum peak excited at the absorption maxima in nm unit. [d]Fluorescence quantum yield using Norharman ($\Phi_{330\sim390}$ = 0.58) as the standard, ± 10%. [e]Fluorescence quantum yield using Rhodamine 6G (Φ_{488} = 0.95) as the standard, ± 10%.

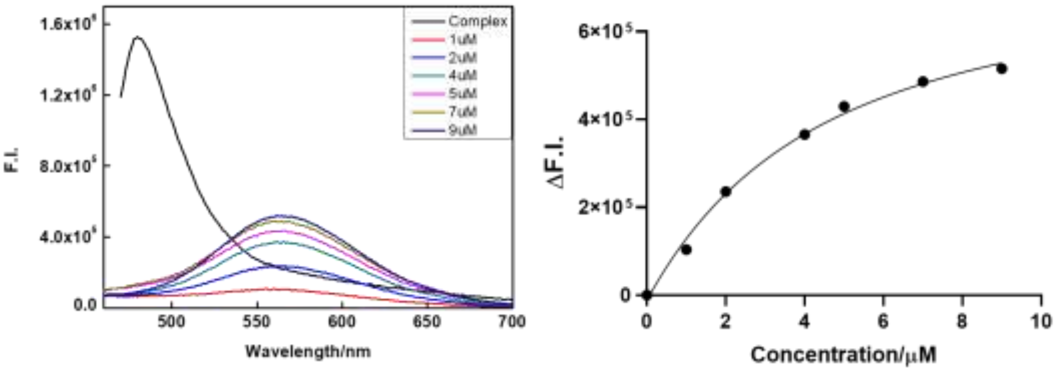

Figure S3. The fluorescence spectra of **R-MA-SLM** (λ_{ex} = 400 nm, c = 10 μM) when adding various amounts of **R-MA-SLM** into the solution of ThT/Aβ_{40} fibrils complex and the corresponding saturation binding curves with various concentrations of **R-MA-SLM**.

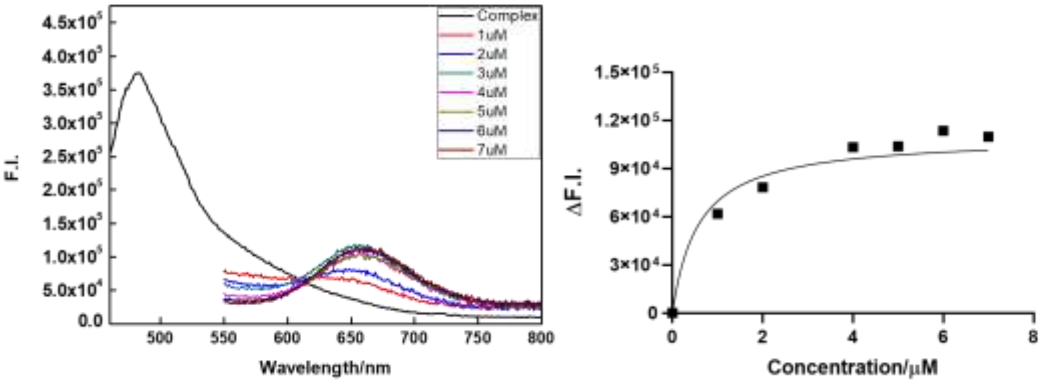

Figure S4. The fluorescence spectra of **MA-SLM** (λ_{ex} = 460 nm, c = 10 μM) when adding various amounts of **MA-SLM** into the solution of ThT/A$\beta_{1\text{-}40}$ fibrils complex

and the corresponding saturation binding curves with various concentrations of **MA-SLM**.

Table S2. The dissociation constants (K_d) of the **R-MA-SLM** and **MA-SLM** with Aβ_{1-40} fibrils.

Compounds	K_d (μM)
**R-MA-SLM**	3.802
**MA-SLM**	0.578

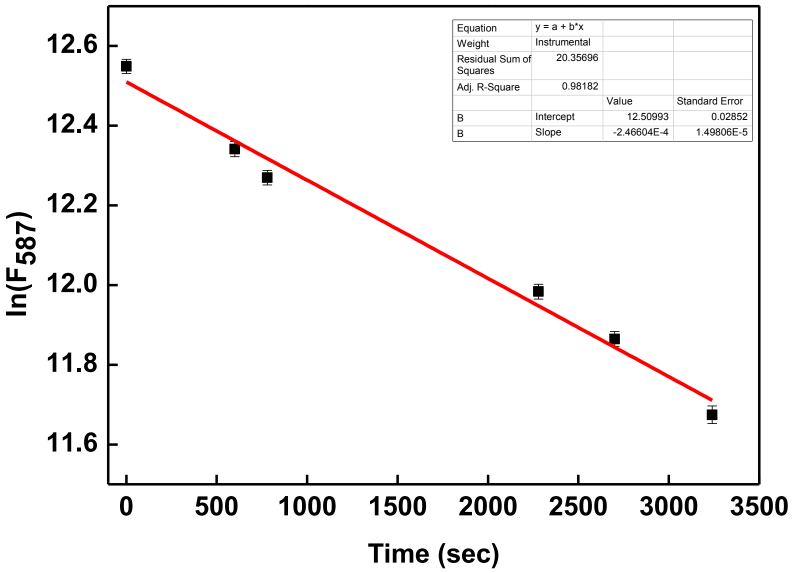

Figure S5. Kinetic plot of the emission intensity change at 587 nm of 20 μM **R-MA-SLM** with addition of 10 μM H_2O_2 with excitation at 490 nm. The red line is the linear fitting of pseudo-first order reaction. The slope of the plot corresponds to the observed reaction rate constant of 2.4 × $10^{-4}s^{-1}$.

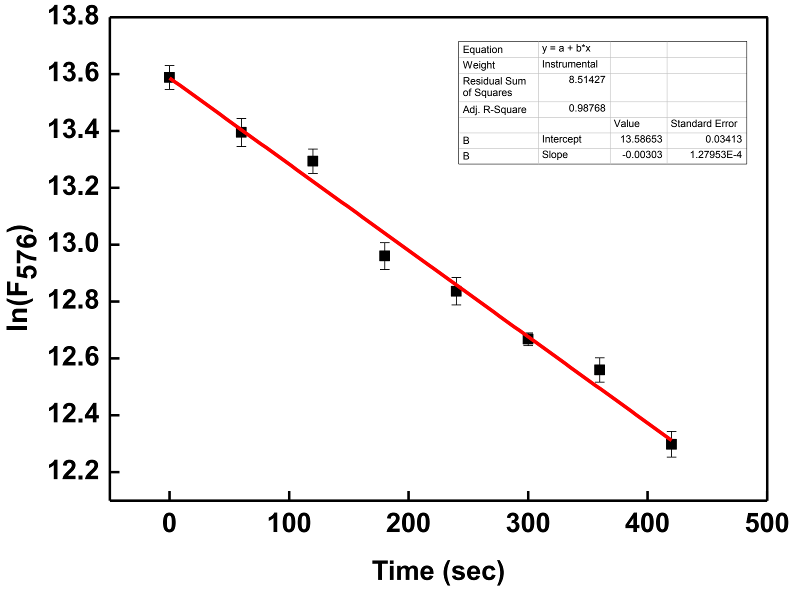

Figure S6. Kinetic plot of the emission intensity change at 576 nm of 20 μM **R-MA-SLM** in the presence of Aβ_{1-40} fibril (10 μM) in PBS with addition of 10 μM H_2O_2 with excitation at 490 nm. The red line is the linear fitting of pseudo-first order reaction. The slope of the plot corresponds to the observed reaction rate constant of 3.0 × $10^{-3}s^{-1}$.

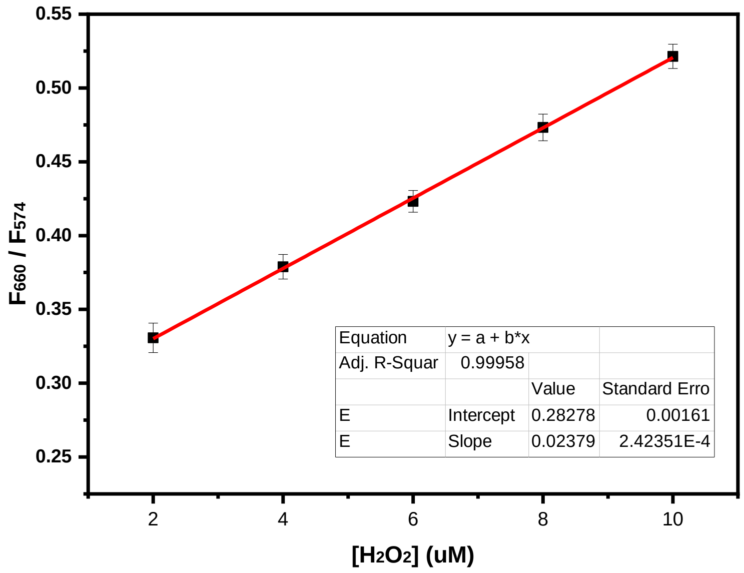

Figure S7. Plot of F_{660}/F_{574} for **R-MA-SLM** (5 μM) vs [H_2O_2] in the range of 2-10 μM in PBS (pH 7.2). The limit of detection (0.26 μM) was calculated with 3σ/k; where σ is the standard deviation of blank measurement; k is the slope.

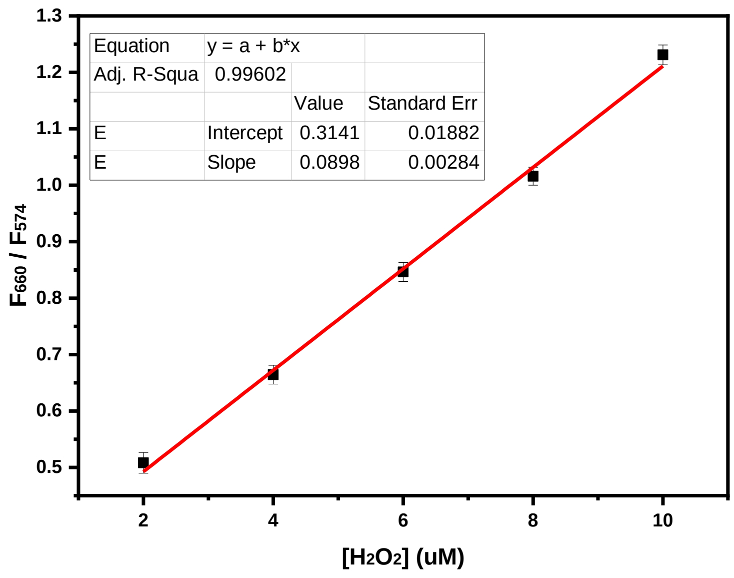

Figure S8. Plot of F_{660}/F_{574} for **R-MA-SLM** (5 μM) in the presence of A$\beta_{1\text{-}40}$ fibril (50 μM) in PBS vs [H_2O_2] in the range of 2-10 μM in PBS (pH 7.2). The limit of detection (0.17 μM) was calculated with 3σ/k; where σ is the standard deviation of blank measurement; k is the slope.

(A)

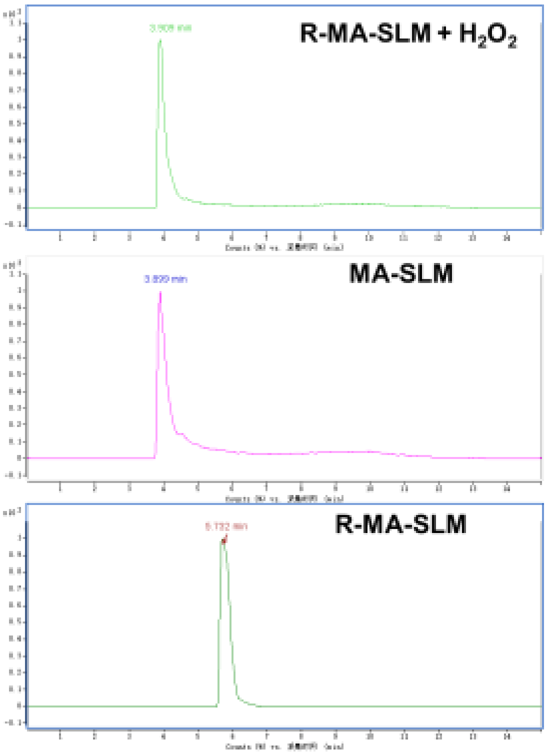

(B)

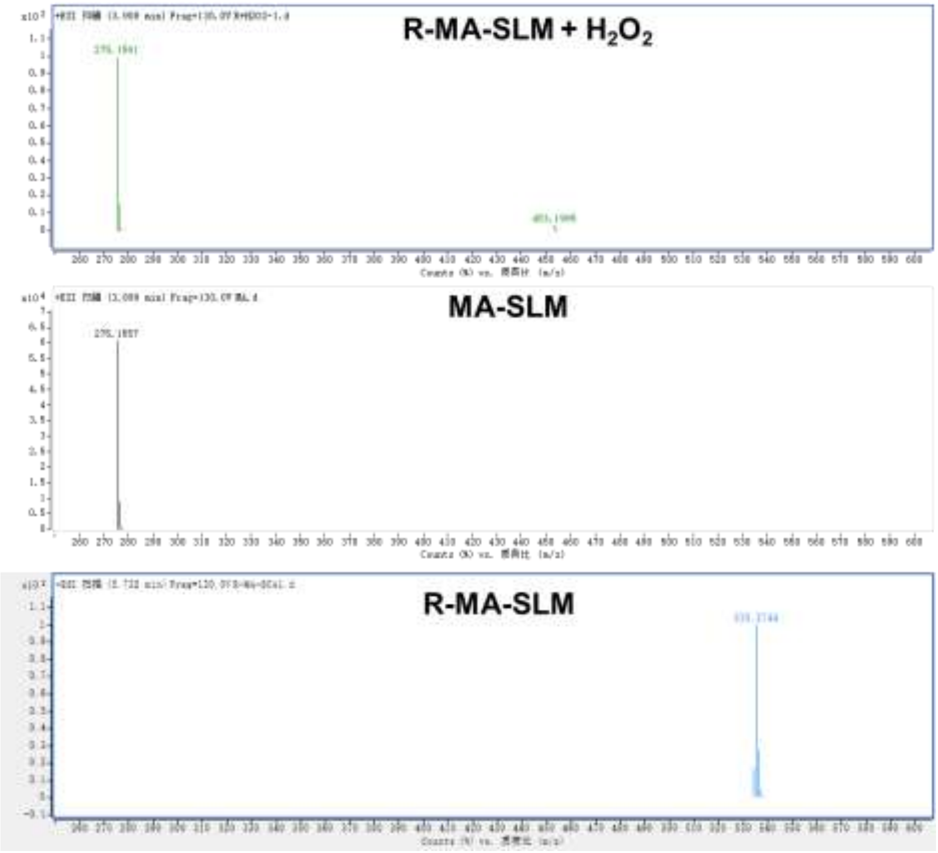

Figure S9. (A) HPLC and (B) HRMS analyses of H_2O_2 treated **R-MA-SLM**, **MA-SLM**, and **R-MA-SLM**.

(A)

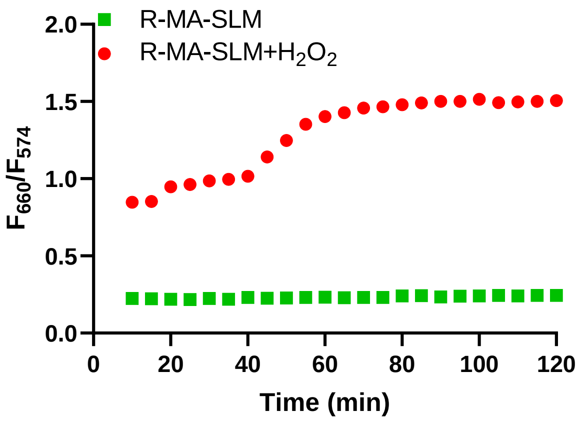

(B)

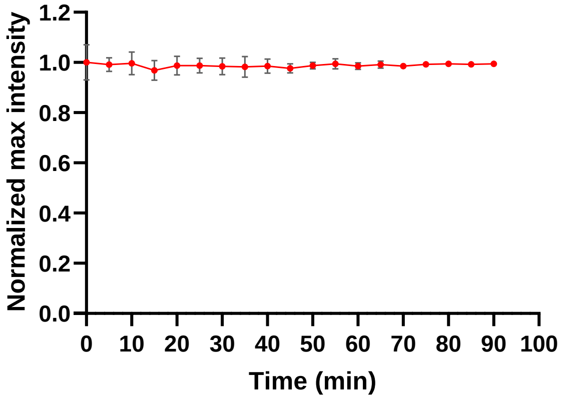

Figure S10. (A) The time courses of fluorescence intensity of **R-MA-SLM** (20 μM) in the presence and absence of H_2O_2 (1 mM). (B) Photostability of the **R-MA-SLM** (50 μM) under ambient light illumination over a period of 90 min at ambient temperature. All the spectra were obtained with the excitation wavelength of 380 nm and recorded the fluorescence intensity at the emission wavelength of 580 nm. Data are expressed as the mean ± SD of three independent measurements (n = 3).

(A)

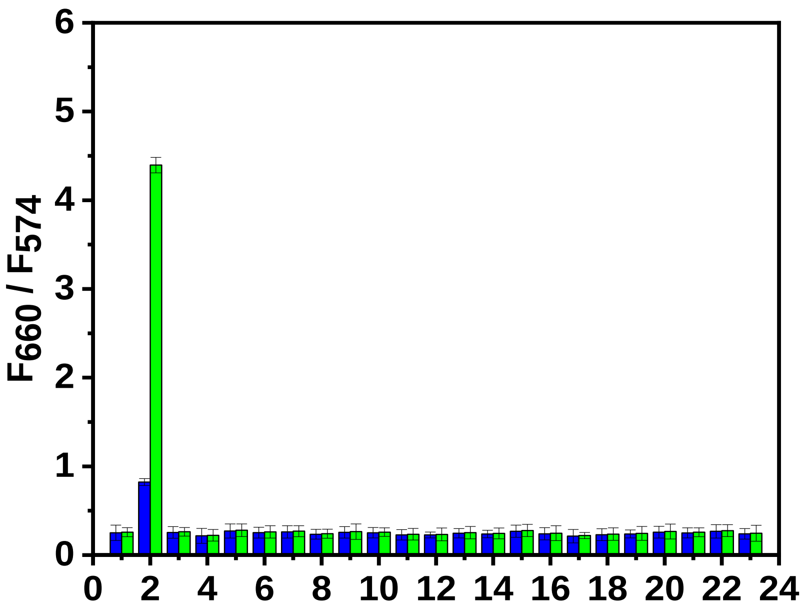

(B)

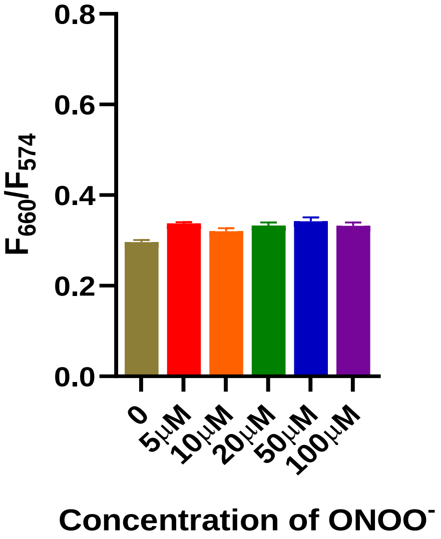

Figure S11. (A) The F_{660}/F_{574} ratio of **R-MA-SLM** (10 μM) in response to various ROS/RNS, metal ions and bioactive small molecules (300 μM) in the absence (blue column) and presence of $A\beta_{1-40}$ fibrils (300 μM) (green column) in PBS after 2 h. 1 to 23 represent blank, H_2O_2, $HO^{\cdot}$, *t*BHP, $\cdot O^tBu$, OCl^-, $^1O_2$, $ONOO^-$, O_2^-, Mg^{2+}, Pb^{2+},

Ca^{2+}, Hg^{2+}, K^{+}, Cu^{2+}, Ag^{+}, Zn^{2+}, Fe^{3+}, Aspartic acid, Cysteine, Phenylalanine, Arginine, Valine, respectively. (λ_{ex} = 490 nm). (B) The F_{660}/F_{574} ratio of R-MA-SLM (10 μM) in response to different concentration of $ONOO^{-}$. Data are expressed as the mean ± SD of three independent measurements (n = 3).

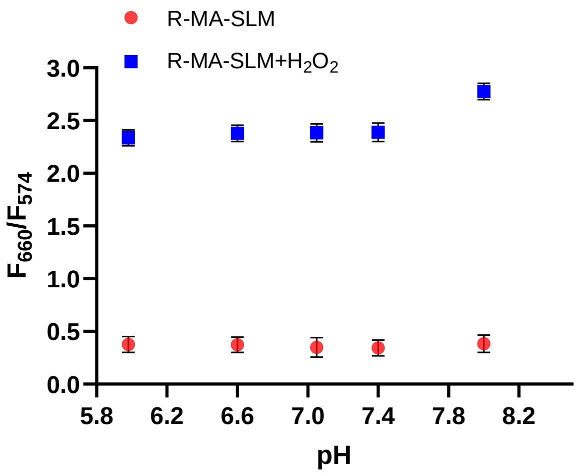

Figure S12. The pH effect on the fluorescence intensity F_{660}/F_{574} ratio of **R-MA-SLM** (20 μM) in the presence and absence of H_2O_2 (200 μM). Data are expressed as the mean ± SD of three independent measurements (n = 3).

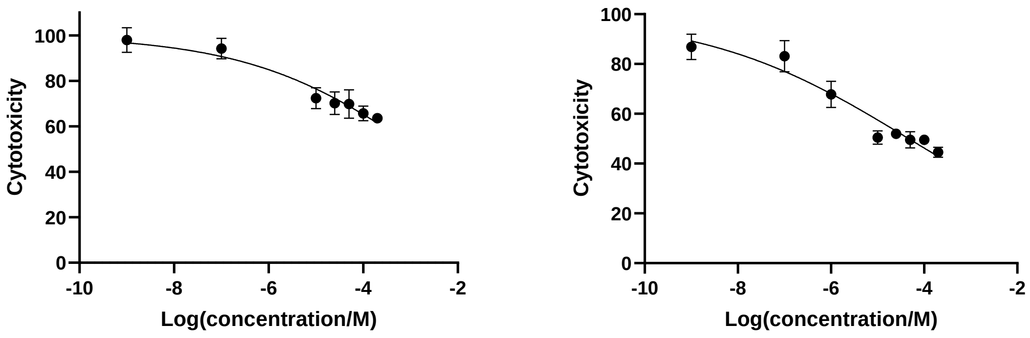

Figure S13. Cell viability values (%) estimated by MTT assay. Human neuroblastoma SH-SY5Y neuronal cells were treated with different concentrations of **R-MA-SLM**

(left) and **MA-SLM** (right) at 37 ˚C for 24 h. Data are expressed as the mean ± SD of three independent measurements (n = 3).

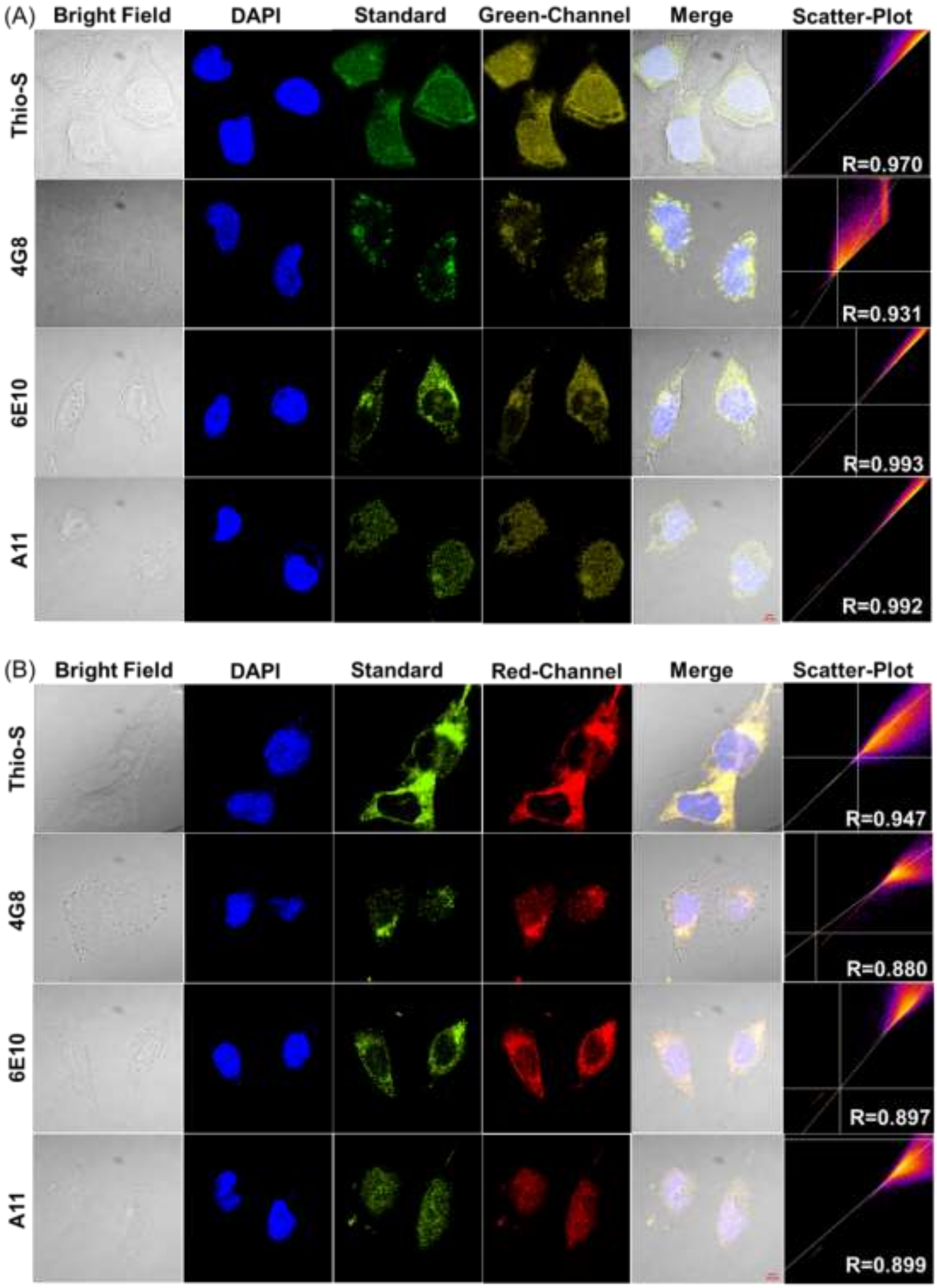

(C)

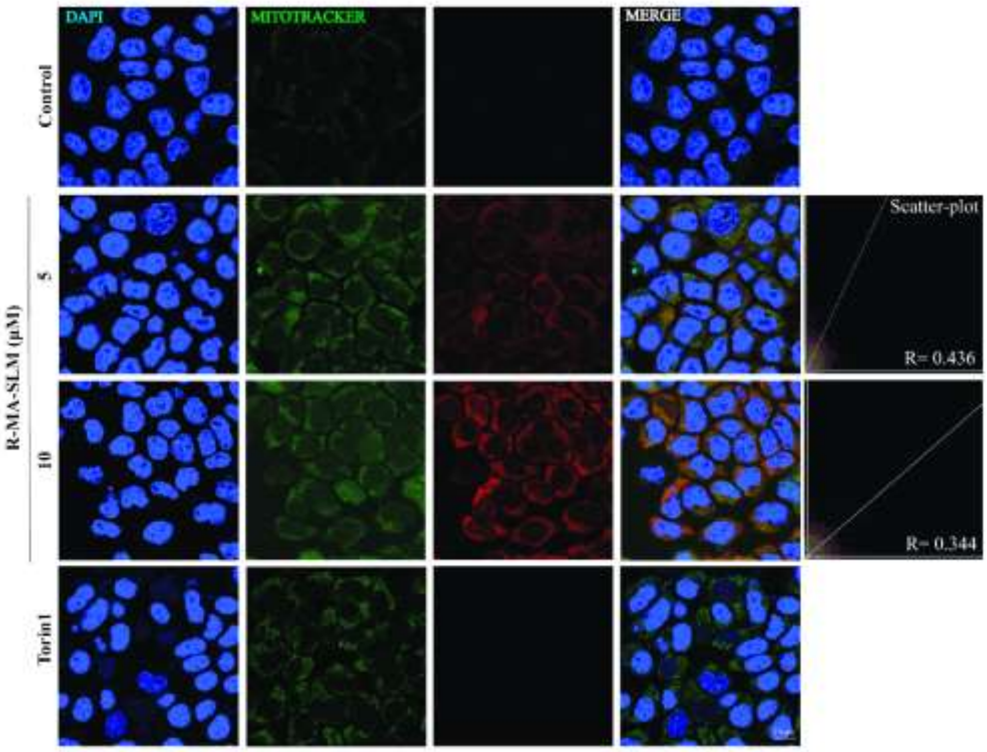

(D)

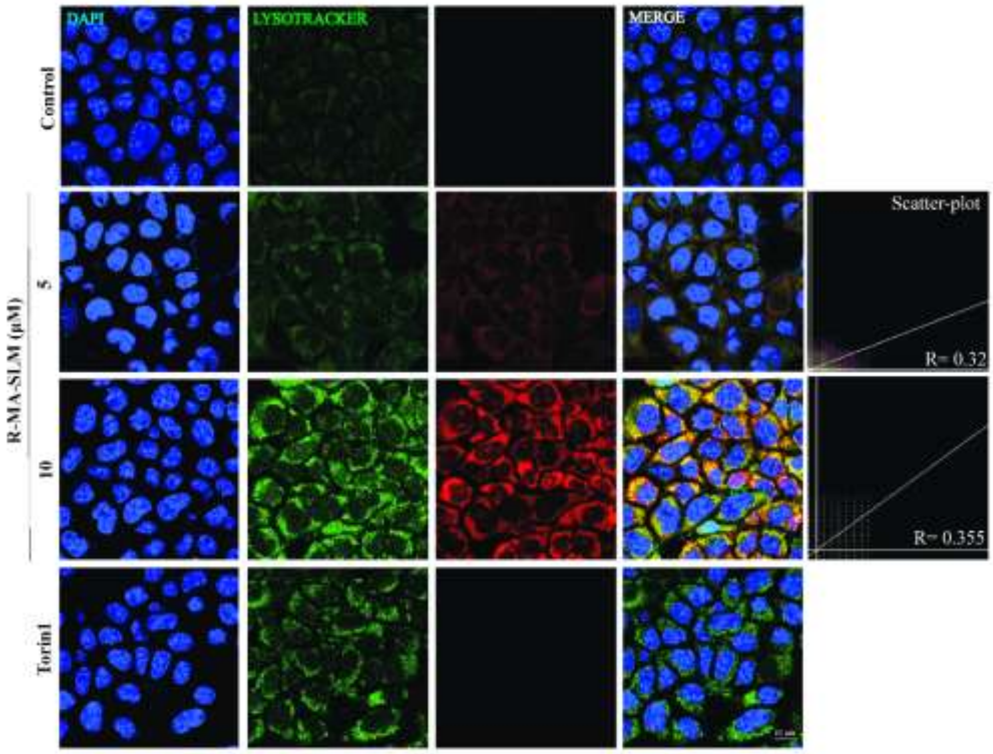

Figure S14. (A) Colocalization analyses of R-MA-SLM dye and immunoreactivities of various Aβ species in N2aSW cells. N2aSW cells were treated with DPI (2.0 μM)

for 15 min and then incubated with R-MA-SLM (20 μM) for 30 mins followed by incubation with Thio-S dye or a primary antibody (6E10, 4G8 or A11) and a secondary antibody conjugated with Alexa 488 as well as their corresponding scatter plots and the average Pearson's colocalization coefficient (R). (B) Colocalization analyses of MA-SLM dye and immunoreactivities of various Aβ species in N2aSW cells. N2aSW cells were treated with DPI (2.0 μM) for 15 min and then incubated with MA-SLM (20 μM) for 30 min followed by incubation with Thio-S dye or a primary antibody (6E10, 4G8 or A11) and a secondary antibody conjugated with Alexa 488 as well as their corresponding scatter plots and the average Pearson's colocalization coefficient (R). Scale bar: 25 μM.

Colocalization analyses of R-MA-SLM dye and organelle trackers in N2a cells, R-MA-SLM (5 and 10 μM) or Torin1 (250 nM) were treated for 24 h and the cells were incubated with (C) LysoTracker Green DND-26 and (D) MitoTracker (50 nM) for the final 1 h. Images of the stained cells were captured by using confocal microscope and their corresponding scatter plots and the average Pearson's colocalization coefficient (R). Scale bar: 10 μm.

(A)

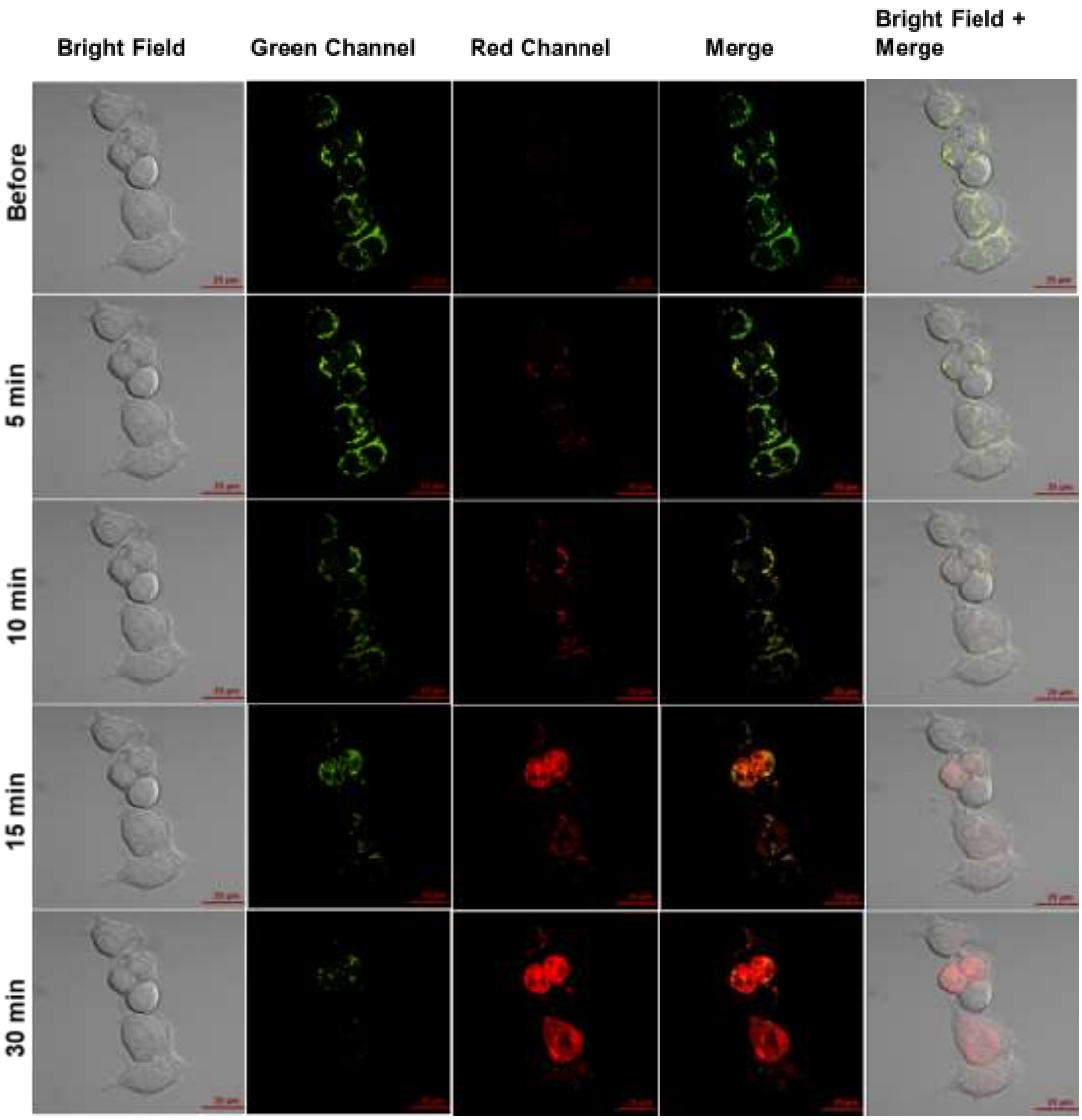
Bright Field
Green Channel
Red Channel
Merge
Bright Field + Merge
Before
5 min
10 min
15 min
30 min

(B)

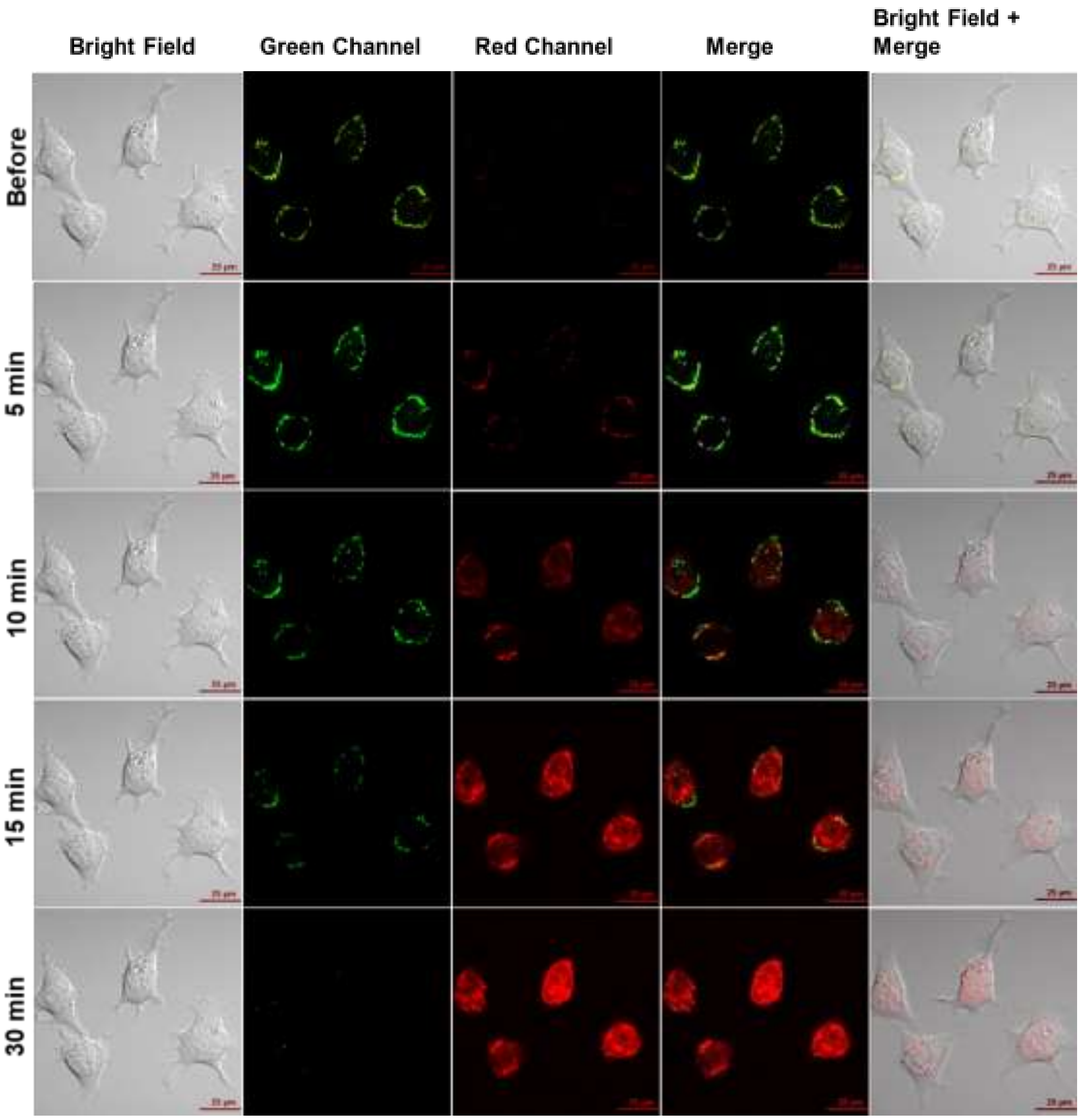

Figure S15. (A) Confocal fluorescence images of N2a cells after being treated with DPI for 15 min and then incubated with **R-MA-SLM** for 5 min. After that, the treated cells were further added with H_2O_2 (20 μM) and the images were recorded from green channel (λ_{em} = 540−600 nm) and red channel (λ_{em} > 650 nm) with an excitation at 490 nm at different time points. (B) Confocal fluorescence images of N2aSW cells after being treated with DPI for 15 min and then incubated with **R-MA-SLM** for 5 min.

After that, the treated cells were further added with H_2O_2 (20 μM). The images were recorded from green channel (λ_{em} = 540−600 nm) and red channel (λ_{em} > 650 nm) at different time points with an excitation at 490 nm.

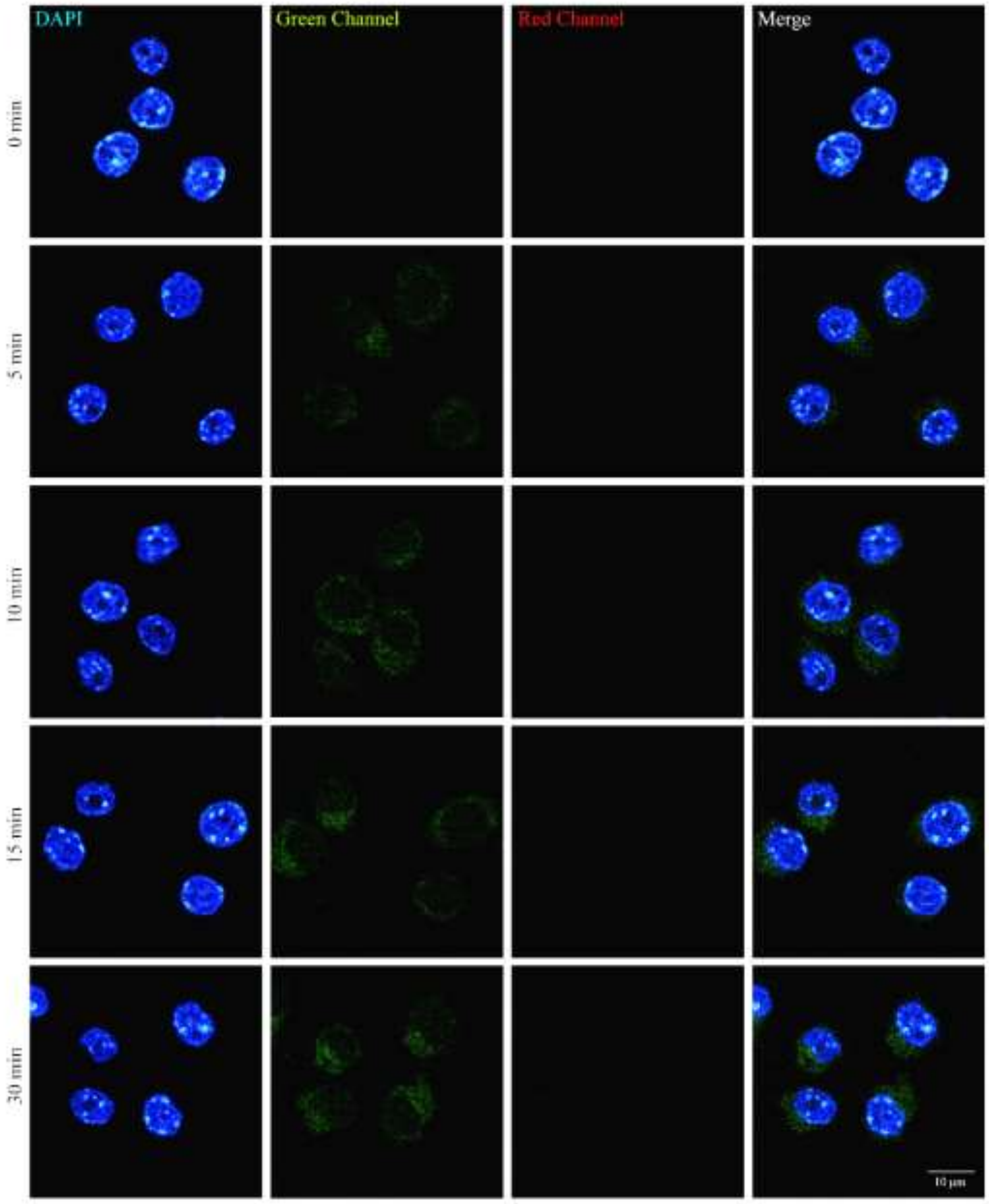

Figure S16. Confocal fluorescence images of immortalized microglial (IMG) cells at different time points after being incubated with R-MA-SLM for 5 min. Fluorescence images were acquired from green channel (λ_{em} = 540−600 nm) and red channel (λ_{em} >

650 nm) with an excitation at 490 nm. DAPI (blue) was used to stain the nucleus. Scale bar: 10 μm.

(A)

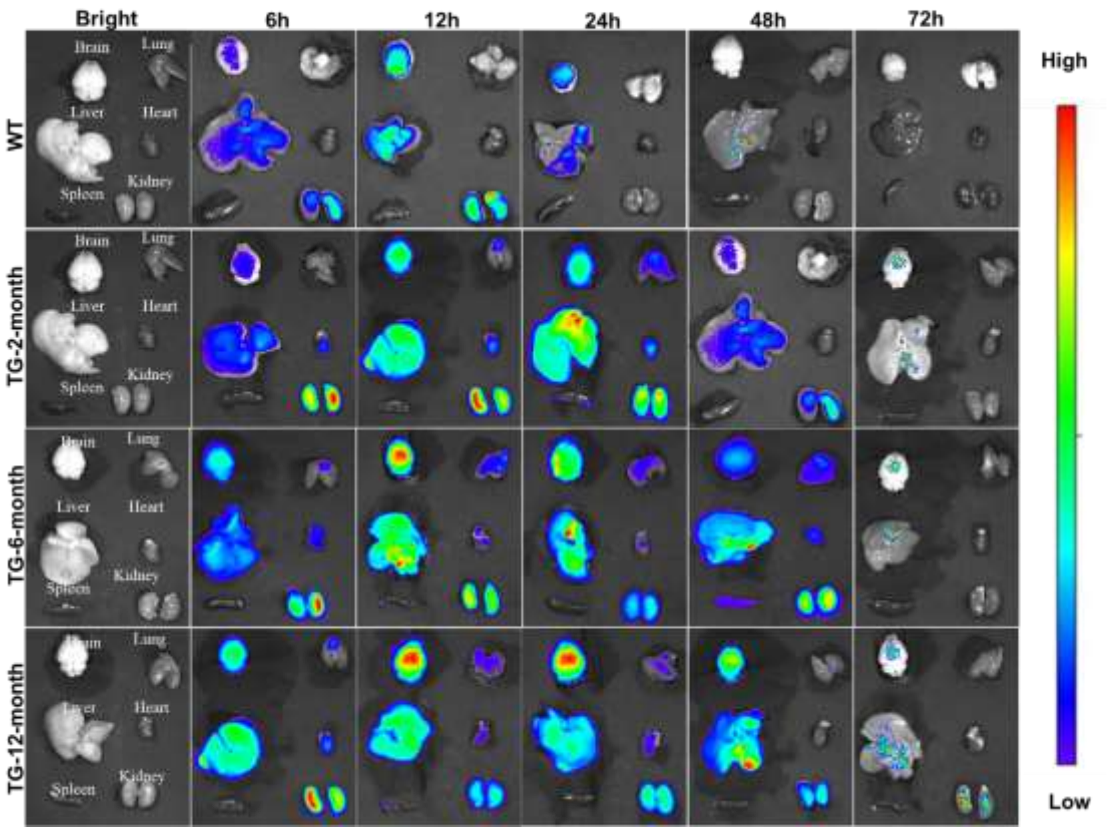

(B)

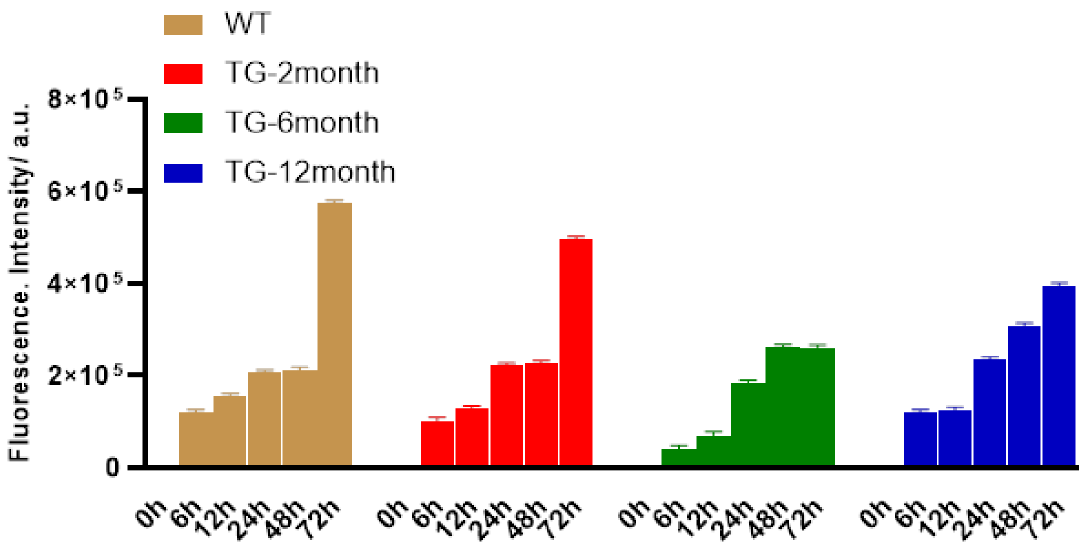

(C)

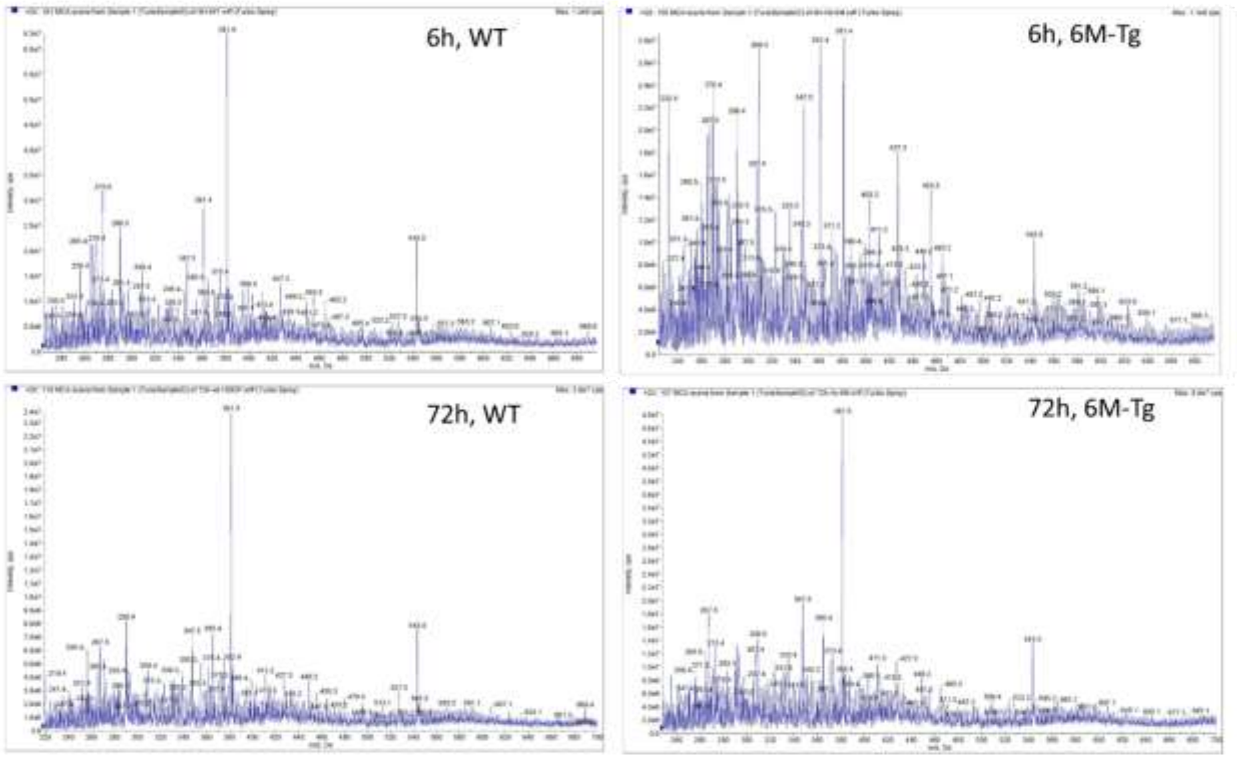

Figure S17. (A) The fluorescence images of **R-MA-SLM** in different organs of 2-month old wild-type and 5XFAD (2-month old, 6-month old and 12-month old) mice acquired from emission at λ_{em} = 575-650 nm with an excitation at λ_{ex} = 500 nm after 6, 12, 24, 48 h and 72 h post-injection of 10 mg/kg **R-MA-SLM**, respectively. (B) The fluorescence intensities of R-MA-SLM of 2-month old wild-type and 5XFAD (2-month old, 6-month old and 12-month old) mice in urine samples at different time points (i.e. 0, 6, 12, 24, 48, and 72 h) before and after post-injection of 10 mg/kg R-MA-SLM at λ_{em} = 541 nm with an excitation at 490 nm. Data are expressed as the mean ± SD of three independent measurements (n = 3). (C) Representative ESI-MS spectra of the urine samples of 2-month old wild-type and 6-month old 5XFAD mice after 6 h and 72 h post-injection of **R-MA-SLM**, respectively.

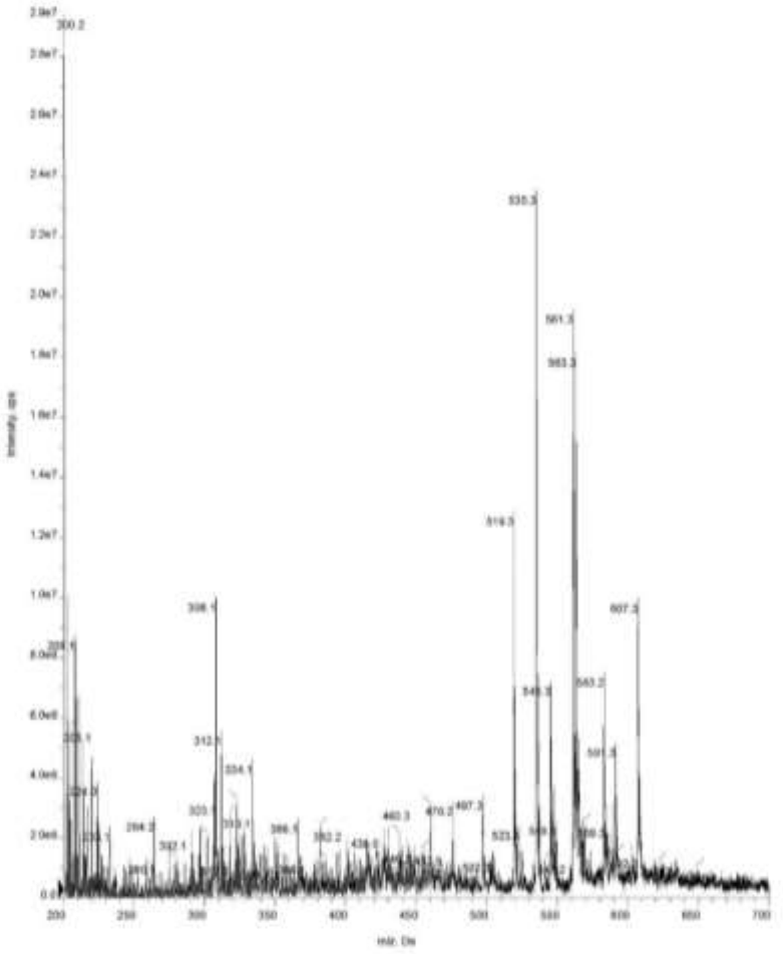

Figure S18. ESI-MS spectrum of the brain extract of the **R-MA-SLM**-treated WT mouse after 6 h post-injection. The maximum content of injection dose of R-MA-SLM in brain and plasma of WT mouse after being administered by intraperitoneal (IP) injection which were estimated to be ~6.5 ng/mg in the brain tissue and ~10.5 ng/100 μL in plasma at 60 min.

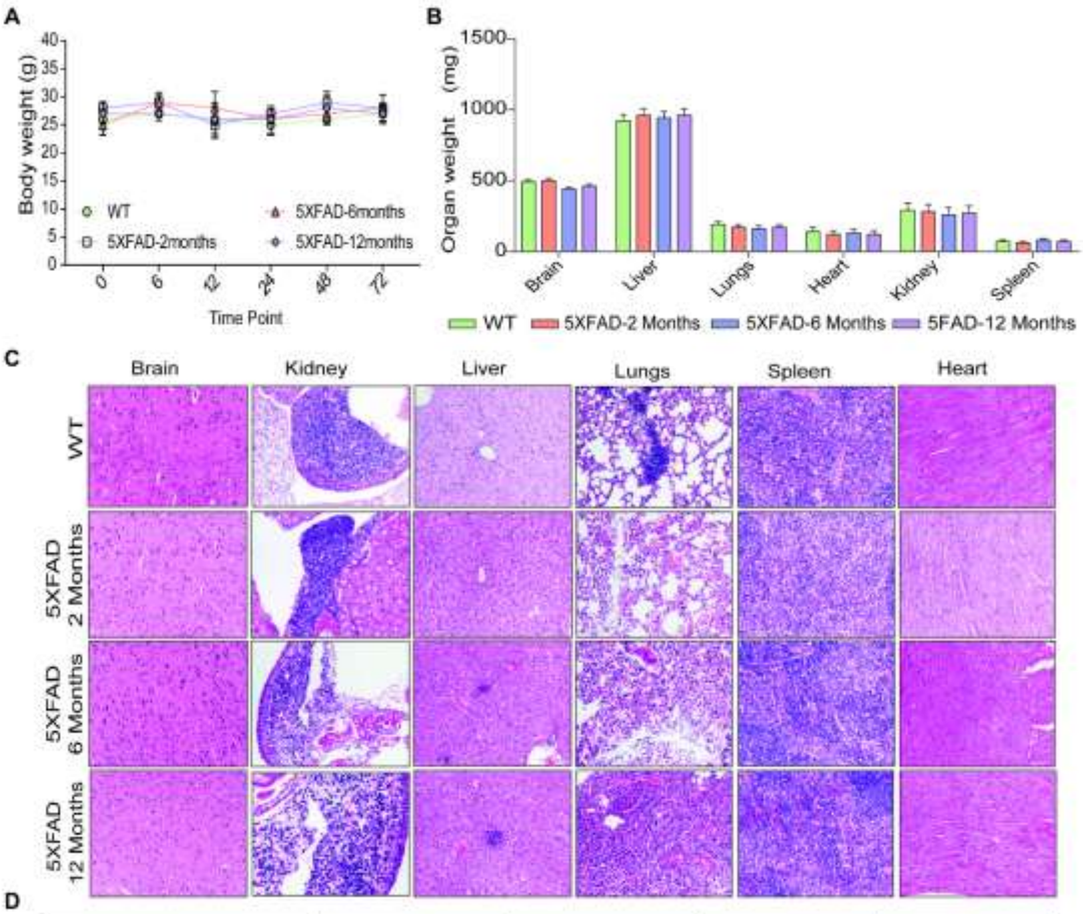

D

Biochemical constituents	WT	5XFAD 2 Months	5XFAD 6 Months	5XFAD 12 Months
ALT/GPT(IU/L)	19.58±7.68	19.17±3.35	19.85±5.79	18.54±9.20
AST/GOT(IU/L)	29.75±7.80	28.33±6.79	29.53±13.76	33.02±10.22
BUN(mM)	17.31±1.20	17.70±3.22	17.10±1.83	18.89±1.71
Cr(μM)	109.56±24.43	110.99±35.25	107.91±13.88	102.49±19.50
T-BIL(μM/L)	29.24±5.32	32.14±0.00	28.13±3.99	29.91±0.00
Na(mM/L)	104.05±4.26	105.45±7.51	108.21±9.28	105.45±8.80
Γ-GT(U/L)	34080.2±2213	35506.2±2213	32719.2±0.00	36506.2±2213
ALB(g/L)	22.49±1.86	23.51±2.53	22.57±2.04	22.92±2.03
ALP(KU/100ml)	7.22±0.86	6.38±1.47	6.97±0.56	6.40±0.89
K(mM)	7.93±0.76	7.75±0.62	8.48±0.35	7.65±0.58
Cl(mM)	70.93±1.47	71.46±3.17	70.94±1.71	70.90±1.56
TC(mM)	2.86±0.63	2.96±0.30	2.57±0.43	2.26±0.75
TG(mM)	0.73±0.13	0.66±0.06	0.76±0.16	0.75±0.25

Figure S19. (A) The body weight of the R-MA-SLM-treated and untreated 5XFAD and WT mice at different time points. (B) The weight of the collected organs from R-MA-SLM-treated 5XFAD mice and the untreated control WT mouse at different time

points. (C) The H&E staining of the different organs of the R-MA-SLM-treated 5XFAD mice and the untreated control WT one. (D) The blood parameter analysis of blood collected in the R-MA-SLM-treated 5XFAD mice and the untreated WT counterpart.

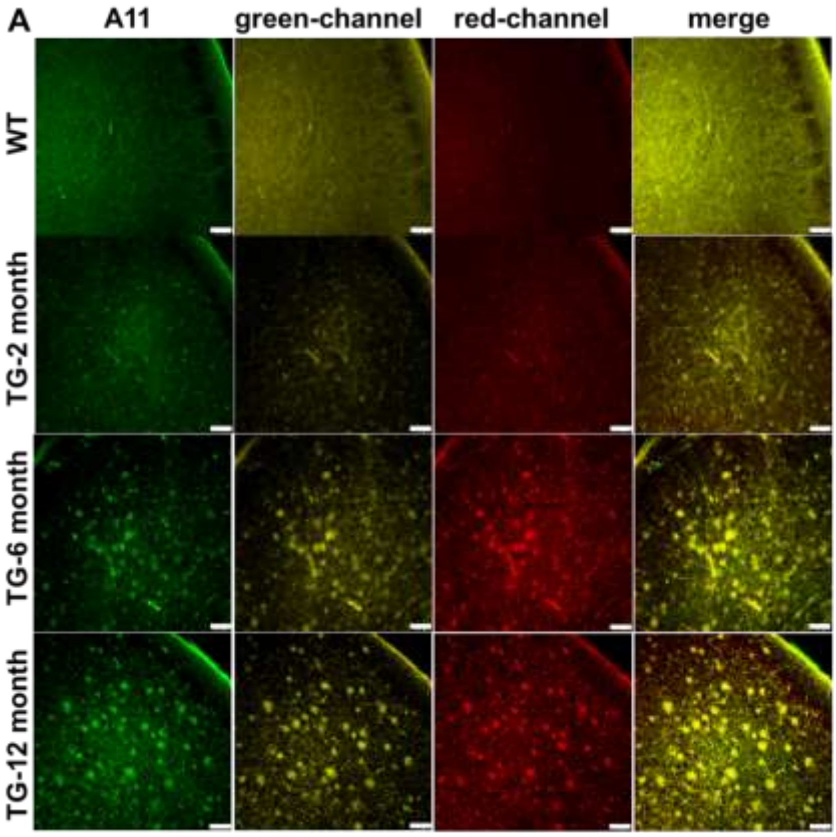

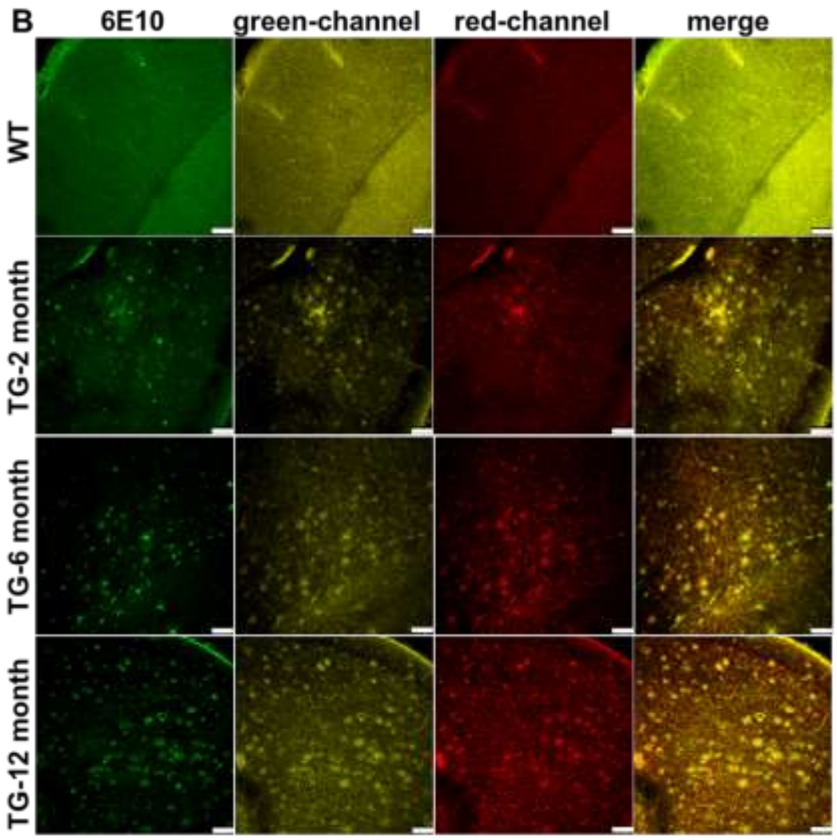
B
6E10
green-channel
red-channel
merge
WT
TG-2 month
TG-6 month
TG-12 month

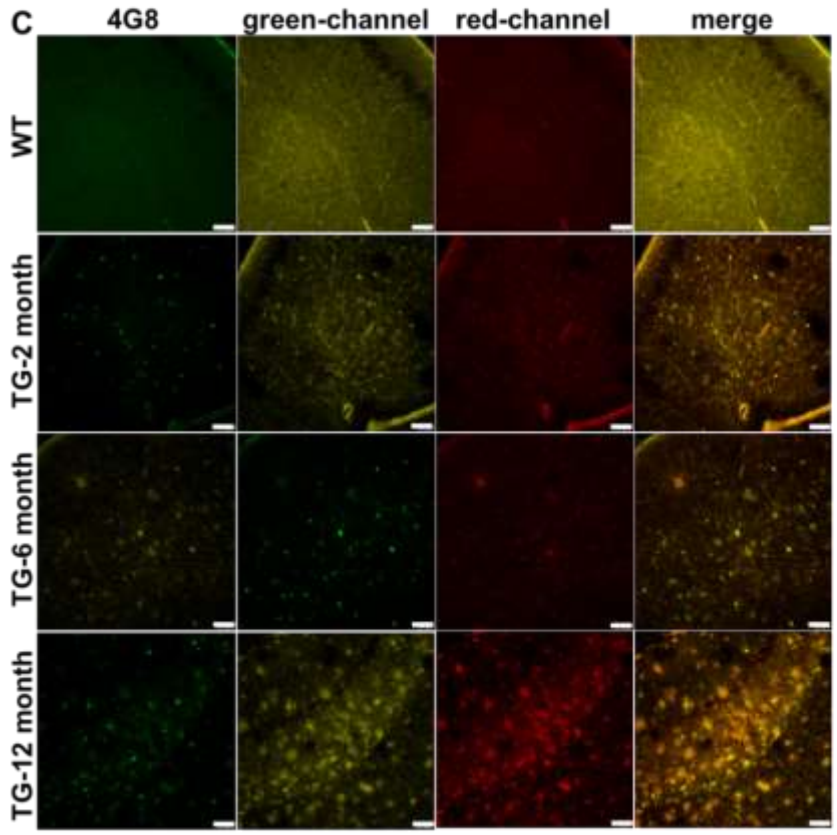
C
4G8
green-channel
red-channel
merge
WT
TG-2 month
TG-6 month
TG-12 month

Figure S20. *Ex vivo* fluorescence images of the brain slices of the WT mouse and 5XFAD mice of different age groups (i.e. 2 months, 6 months and 12 months) after injection of **R-MA-SLM** (20 mg/kg) for 60 min, followed by co-staining with a primary antibody, (A) A11, (B) 6E10, and (C) 4G8. λ_{ex} = 488 nm, λ_{em} = 540-600 nm (green-channel); λ_{em} = 650-700 nm (red-channel). Scale bar: 100 μm.

Table S3. Summary of Reported Fluorescence Probes for Imaging of ROS in Alzheimer's Disease.

Probe	Detection principle	λ^{em}_{max} (nm)[a]	λ^{em}_{max} with ROS (nm)[b]	Type of ROS[c]	Quantitative detection with LOD for ROS[d]	Cell model imaging	Ex vivo imaging of brain sections	In vivo imaging of mouse model	Mouse model & age (months)
R-MA-SLM[1]	ratiometric	574	661	H_2O_2	yes (0.17 μM)	N2aSW	yes	yes	5XFAD (2, 6,12)
CRANAD-61[2]	dual-color	810	570	Non-specific	no	no	yes	yes	APP/PS1 (4,12,18)
CM2[3]	turn-on	-	485	HOCl	no	SHSY5Y	yes	no	APP/PS1 (10)
CRANAD-88[4]	fluorescence	690	730	H_2O_2	no	no	Yes (upon addition of H_2O_2)	yes	APP/PS1 (15)

[a]Emission maximum excited at the absorption maximum. [b]Emission maximum excited at the absorption maximum upon ROS reaction. [c] Type of ROS that the probe specifically responds to. [d]Limit of detection (LOD) in the presence of Aβ.

[1]The present work.
[2]Yang, J.; Zhang, X.; Yuan, P.; Yang, J.; Xu, Y.; Grutzendler, J.; Shao, Y.; Moore, A.; Ran, C., Oxalate-curcumin-based probe for micro- and macroimaging of reactive oxygen species in Alzheimer's disease. *Proc Natl Acad Sci U S A* **2017,** *114* (47), 12384-12389.
[3]Samanta, S.; Govindaraju, T., Unambiguous Detection of Elevated Levels of Hypochlorous Acid in Double Transgenic AD Mouse Brain. *ACS Chem Neurosci* **2019,** *10* (12), 4847-4853.
[4]Yang, J.; Yang, J.; Liang, S. H.; Xu, Y.; Moore, A.; Ran, C., Imaging hydrogen peroxide in Alzheimer's disease via cascade signal amplification *Sci. Rep.* **2016**, *6*, 35613.
